# Single high-fat challenge and trained innate immunity: A randomized controlled cross-over trial

**DOI:** 10.1016/j.isci.2024.111103

**Published:** 2024-10-05

**Authors:** Julia van Tuijl, Julia I.P. van Heck, Harsh Bahrar, Wieteke Broeders, Johan Wijma, Yvonne M. ten Have, Martin Giera, Heidi Zweers-van Essen, Laura Rodwell, Leo A.B. Joosten, Mihai G. Netea, Lydia A. Afman, Siroon Bekkering, Niels P. Riksen

**Affiliations:** 1 Department of Internal Medicine, Radboud University Medical Center Nijmegen, Nijmegen 6525 GA, the Netherlands; 2 Center for Proteomics and Metabolomics, Leiden University Medical Center, Leiden 2333 ZA, the Netherlands; 3 Department of Gastroenterology and Hepatology-Dietetics, Radboud University Medical Center, Nijmegen 6525 GA, the Netherlands; 4 Section Biostatics, Department of Health Evidence, Radboud University Medical Center, Nijmegen 6525 GA, the Netherlands; 5 Department of Medical Genetics, Iuliu Haţieganu University of Medicine and Pharmacy, 400347 Cluj-Napoca, Romania; 6 Department for Immunology & Metabolism, Life and Medical Sciences Institute (LIMES), University of Bonn, 53115 Bonn, Germany; 7 Nutrition, Metabolism and Genomics Group, Division of Human Nutrition and Health, Wageningen University and Research, Wageningen 6700 HB, the Netherlands; 8 Murdoch Children’s Research Institute, Royal Children’s Hospital, Melbourne, VIC 3052, Australia

**Keywords:** Health sciences, Human Physiology, Human metabolism

## Abstract

Brief exposure of monocytes to atherogenic molecules, such as oxidized lipoproteins, triggers a persistent pro-inflammatory phenotype, named trained immunity. In mice, transient high-fat diet leads to trained immunity, which aggravates atherogenesis. We hypothesized that a single high-fat challenge in humans induces trained immunity. In a randomized controlled cross-over study, 14 healthy individuals received a high-fat or reference shake, and blood was drawn before and after 1, 2, 4, 6, 24, and 72 h. Incubation of donor monocytes with the post-high-fat-shake serum induced trained immunity, regulated via Toll-like receptor 4. This was not mediated via triglyceride-rich lipoproteins, C12, 14, and 16, or metabolic endotoxemia. *In vivo*, however, the high-fat challenge did not affect monocyte phenotype and function. We conclude that a high-fat challenge leads to alterations in the serum composition that have the potential to induce trained immunity *in vitro*. However, this does not translate into a (persistent) hyperinflammatory monocyte phenotype *in vivo*.

## Highlights

• Human post-high-fat-load serum can induce trained immunity in monocytes *in vitro*
• This is mediated via TLR4, and not by lipoproteins, C12/14/16, or by LPS
• Isolated PBMCs 3 days after high-fat load do not show inflammatory changes
• Metabolic effects of high-fat load are too short to induce trained immunity *in vivo*

## Graphical abstract

**Figure:**
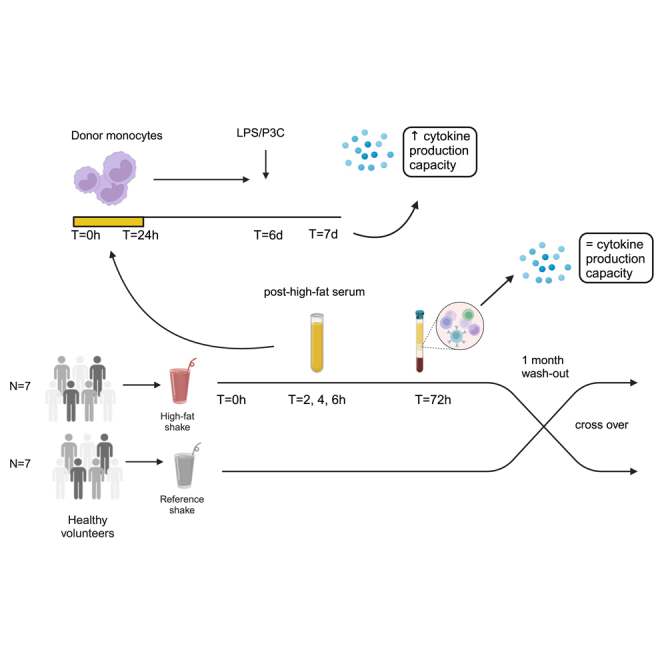

## Introduction

Over the past few decades, improved health care, hygiene, and food resources have rapidly increased human life expectancy. The additional years that these innovations have added to our lives, however, do not always account for a longer *healthy* lifespan. The modern Western lifestyle, including consumption of a Western-type diet (WTD), contributes to the increased prevalence of chronic non-communicable diseases. Among these diseases, cardiovascular disease (CVD) has emerged as one of the leading contributors to the loss of healthy life years.^1^

A Western lifestyle is associated with obesity, dyslipidemia, and insulin resistance, which can potentially induce (systemic) inflammatory processes driving atherosclerosis. Whereas research in CVD has long been focused on low-density lipoprotein (LDL) and high-density lipoprotein (HDL), genetic Mendelian randomization studies have now convincingly demonstrated that triglyceride (TG)-rich lipoproteins (TRLs) are also causally associated with inflammation and CVD.^2^ In the Copenhagen City Heart Study and Copenhagen General Population Study, high concentrations of nonfasting TGs were associated with an augmented risk of atherosclerotic CVD.^2^ Human studies have shown that the postprandial hypertriglyceridemia, occurring after the ingestion of a high-fat meal, can mediate systemic increases of a wide range of inflammatory factors, including a brief elevation of circulating lipopolysaccharide (LPS) (metabolic endotoxemia), and an activation of circulating monocytes.^3^^,^^4^^,^^5^^,^^6^^,^^7^^,^^8^

Since most high-fat challenge studies in humans have only explored the acute phase after ingestion of a high-fat meal, it remains elusive whether these transient postprandial changes can contribute to the chronic low-grade inflammation, which characterizes atherosclerosis. In contrast to the traditional immunological paradigm, it has recently been established that cells of the innate immune system such as monocytes or macrophages, can also build an immunological memory. This can be induced by microbial stimuli, but also by endogenous atherogenic stimuli, such as oxidized low-density lipoprotein and lipoprotein(a).^9^^,^^10^^,^^11^^,^^12^^,^^13^ This innate immune memory has been named *trained immunity* and is mediated by a rapid transcriptional, metabolic, and epigenetic reprogramming, which results in a persistent augmentation of immune effector functions, with cytokine production capacity as the main readout.^14^ Key metabolic changes driving trained immunity include activation of glycolysis, glutaminolysis, and the mevalonate pathway. Intermediate metabolites of these pathways subsequently induce long-term epigenetic modifications, including an enrichment of histone 3 lysine 4 mono- and trimethylation.^14^ The clinical relevance of trained immunity in the development of atherosclerosis in humans has been suggested by several recent observational studies. In patients with established coronary artery disease and in patients with familial hypercholesterolemia, circulating monocytes showed an enhanced cytokine production capacity, associated with changes in their epigenetic and metabolic signatures.^15^^,^^16^ In a murine model of atherosclerosis, a 4-week WTD can evoke a persistent activation of the innate immune system, by transcriptional and functional reprogramming of their bone marrow progenitor cells, which persisted even 4 weeks after reversing to a regular diet.^17^ In addition, repetitive systemic administration of very low dosages of LPS accelerates atherosclerosis by trained immunity.^18^

We thus hypothesize that the temporary changes in lipoproteins and/or endotoxemia following high-fat meals contribute to atherosclerosis via the induction of trained immunity. To test our hypothesis, we exposed healthy volunteers to a single high-fat challenge and collected blood and serum at different time points thereafter. We exposed healthy human monocytes to the serum obtained after the high-fat load in order to identify its capacity to induce *trained immunity*. In addition, we examined the *ex vivo* cytokine production capacity and monocyte subsets and activation markers from the high-fat-exposed subjects at early and late time points.

## Results

### Subject characteristics

A total of 19 participants were screened for eligibility. Three participants were excluded of which one did not meet the inclusion criteria and two declined to participate. Sixteen participants underwent randomization (see flow diagram in Figure S1). Eight participants first received the high-fat challenge, and 8 participants first received the control shake. In each study arm, there was one dropout after the first study visit. Therefore, 14 participants in total completed all study days for both of the shakes. Baseline characteristics of the participants are listed in Table 1.

**Table 1:** Baseline characteristics of participants

Baseline characteristics of participants (*n* = 14)	
Sex (% male)	50%
Age (years)	24 ± 4
Race, white (%)	100%
Body mass index (kg/m^2^)	23.3 ± 2.6
Waist circumference (cm)	78 ± 9
Systolic blood pressure (mmHg)	126 (108–158)
Diastolic blood pressure (mmHg)	73 (64–90)
Glucose (mmol/L)	4.9 ± 0.7
Total cholesterol (mmol/L)	4.14 ± 0.62
LDL cholesterol (mmol/L)	2.21 ± 0.67
HDL cholesterol (mmol/L)	1.52 ± 0.38
Non-HDL cholesterol (mmol/L)	2.64 ± 0.69
Triglycerides (mmol/L)	0.96 ± 0.41

Data are presented as mean ± SD when normally distributed or as median (with minimum and maximum values) for non-normally distributed continuous variables, and as percentages for categorical data.

### Postprandial metabolic response to the different shake types

**Figure 1:**
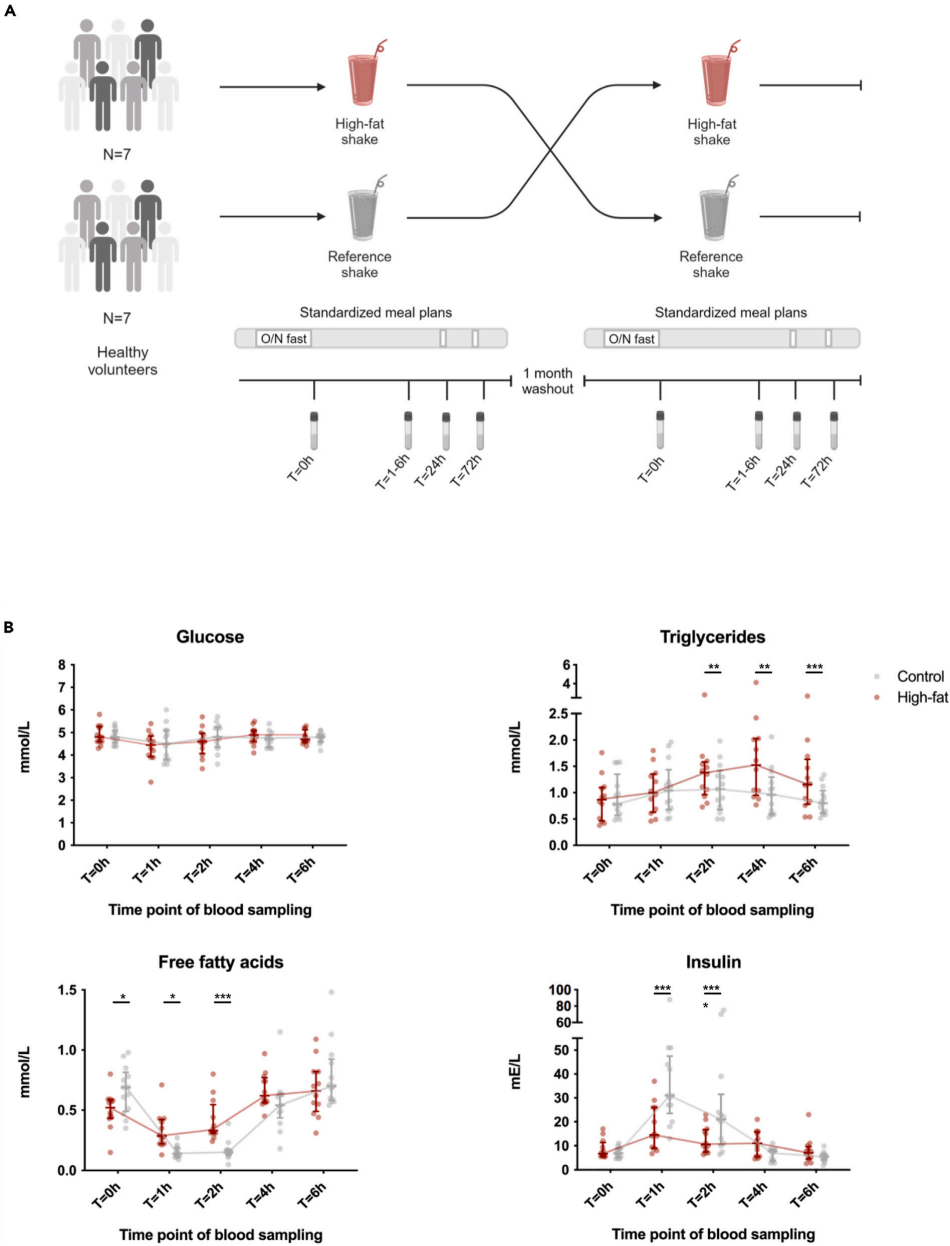
Study design and postprandial metabolite changes (A) Graphical outline of the *in vivo* study design. A total of 14 participants received a high-fat shake and a reference shake in a cross-over design. Seven participants first received the high-fat shake, and seven participants first received the reference shake. To exclude carryover effects, the participants received the other shake after a washout period of at least 1 month. Blood was drawn before (t = 0 h) and at several time points after the consumption of both shakes (t = 1 h, t = 2 h, t = 4 h, t = 6 h, t = 24 h, t = 72 h). Starting from the evening before the ingestion of the shake until time point t = 72 h, the participants were provided with standardized meal plans. See also Figure S1. Created with BioRender.com. (B) At t = 0 h, t = 1 h, t = 2 h, t = 4 h, and t = 6 h glucose, triglyceride, free fatty acid, and insulin concentrations were measured for each shake (*n* = 14). Median ± IQR. ∗ indicates two-sided *p* < 0.05, ∗∗*p* < 0.01, ∗∗∗*p* < 0.001, ∗∗∗∗*p* < 0.0001; no sign indicates no significant differences, as calculated with linear mixed model for repeated measures. Due to technical errors, data from 1 participant was missing for the control shake and from 1 participant for the high-fat shake. Missing data were fitted with the restricted maximum likelihood approach.

Baseline and postprandial concentrations of circulating TGs, free fatty acids (FFAs), glucose, and insulin are shown in Figure 1B. A significant difference in response between the high-fat shake and the reference shake was observed for TG at t = 2 h (*p* = 0.034), t = 4 h (*p* = 0.00001), and t = 6 h (*p* = 0.0002); for FFA at t = 0 h (*p* = 0.038), t = 1 h (*p* = 0.0125), and t = 2 h (*p* = 0.0001); and for insulin at t = 1 h (*p* = 0.00001) and t = 2 h (*p* = 0.0002), reflecting the higher fat content at the expense of carbohydrate content in the high-fat shake. The shake × time interaction was significantly different (meaning the change within the intervention period is different between the shakes) for TG (*p* < 0.001), for FFA (*p* < 0.001), and for insulin (*p* < 0.0001). The time effect was significant for all four metabolites (*p* < 0.001 for all). The shake effect was significant for TG (*p* < 0.001), FFA (*p* < 0.001), and insulin (*p* < 0.001). There was no significant effect of period, which excludes carryover effects.

### Serum isolated after a high-fat challenge induces trained immunity in human monocytes

**Figure 2:**
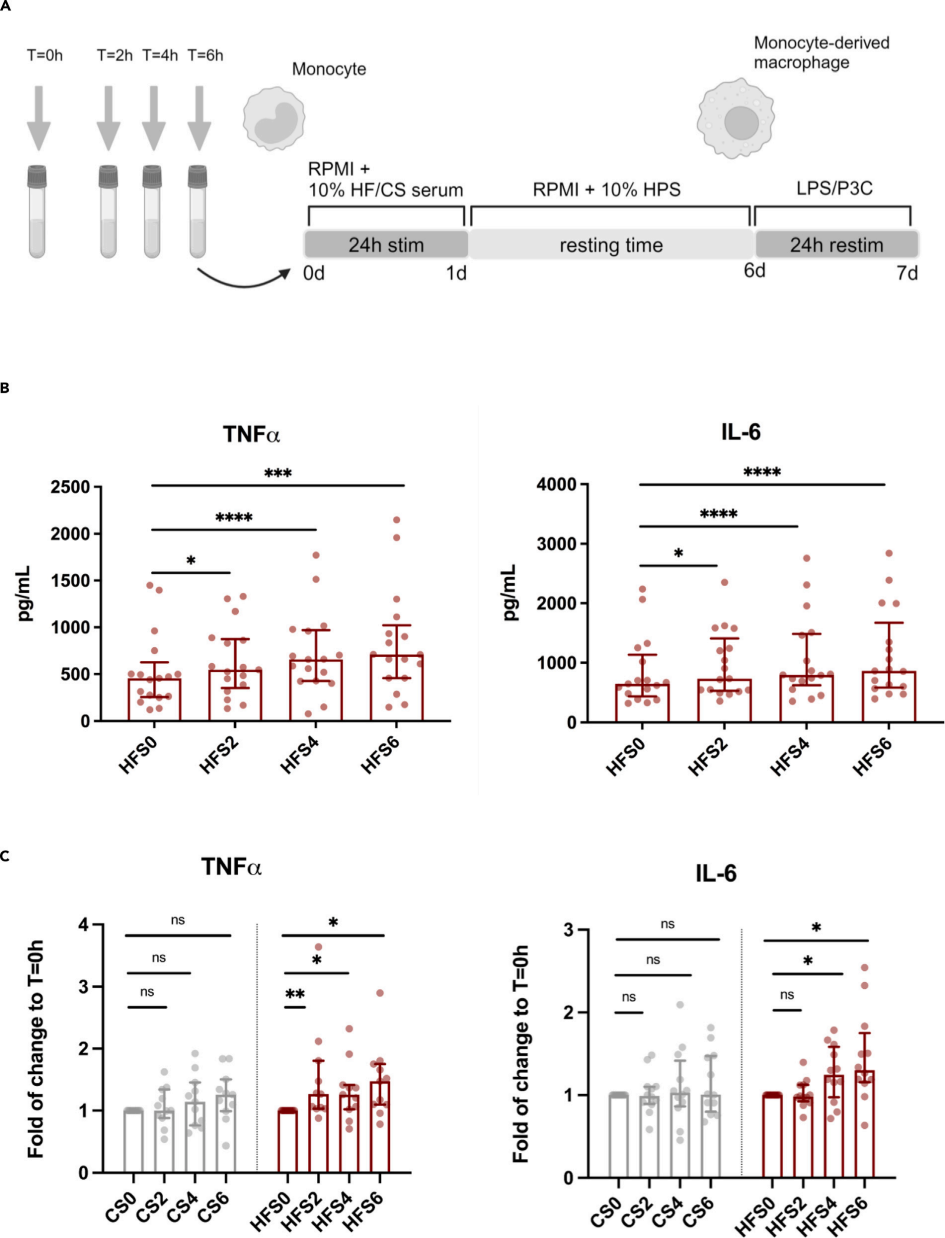
Exposure to serum obtained after a high-fat shake, but not after a common breakfast shake, induces trained immunity in healthy human monocytes (A) Graphical outline of the design of the *in vitro* experiments. Adherent healthy human monocytes were exposed to serum obtained before (t = 0 h; CS0/HFS0) and at several time points after (t = 2 h [CS2/HFS2], t = 4 h [CS4/HFS4], and t = 6 h [CS6/HFS6]) consumption of either the reference or high-fat shake. After 24 h incubation time, cells were rested and differentiated into macrophages. On day 6, the cells were restimulated with Toll-like receptor (TLR)-agonists (TLR4; lipopolysaccharide [LPS] and TLR2; Pam3Cys [P3C]) for another 24 h before cytokine production was measured with ELISA. Created with BioRender.com. (B) Serum obtained after the high-fat shake increased TNF-α and IL-6 production of human monocyte-derived macrophages upon secondary stimulation with LPS, with the highest cytokine production after stimulation with serum obtained at t = 6 h (*n* = 17). (C) Cytokine production capacity was measured after 24 h exposure to reference shake serum (CS) and the high-fat shake serum (HFS). Data are presented as fold of change to the fasting serum obtained before consumption of the control shake (CS0) or the high-fat shake (HFS0) (*n* = 11). Median ± IQR. ∗ indicates two-sided *p* < 0.05, ∗∗*p* < 0.01, ∗∗∗*p* < 0.001, ∗∗∗∗*p* < 0.0001, Wilcoxon signed-rank test. CS, serum after the reference shake; HFS, serum after the high-fat shake. See also Figure S2.

We obtained serum at baseline and at t = 2, 4, and 6 h after consumption of the shakes from all 14 participants. After exclusion of 1 participant (due to signs of an infectious disease), we pooled the high-fat shake sera from 13 participants for each separate time point to use in *in vitro* experiments. The same was done for the reference shake sera. Adherent human monocytes from healthy donors were transiently exposed to the sera for 24 h and stimulated again with Toll-like receptor (TLR) 4 and 2 ligands, LPS, and Pam3Cys (P3C) on day 6. Twenty four hour exposure of monocytes to the pooled postprandial serum obtained after the high-fat shake induced increased production of tumor necrosis factor α (TNF-α) and interleukin (IL)-6 upon secondary stimulation compared to monocytes exposed to fasting serum (Figure 2B). Stimulation of monocytes with pooled postprandial serum obtained after the reference shake did not induce any significant cytokine production upon secondary stimulation (Figure 2C). Results upon restimulation with P3C can be found in Figure S2.

### High-fat challenge-induced trained immunity occurs via TLR4 but is not mediated by TRLs, FFAs, or metabolic endotoxemia

**Figure 3:**
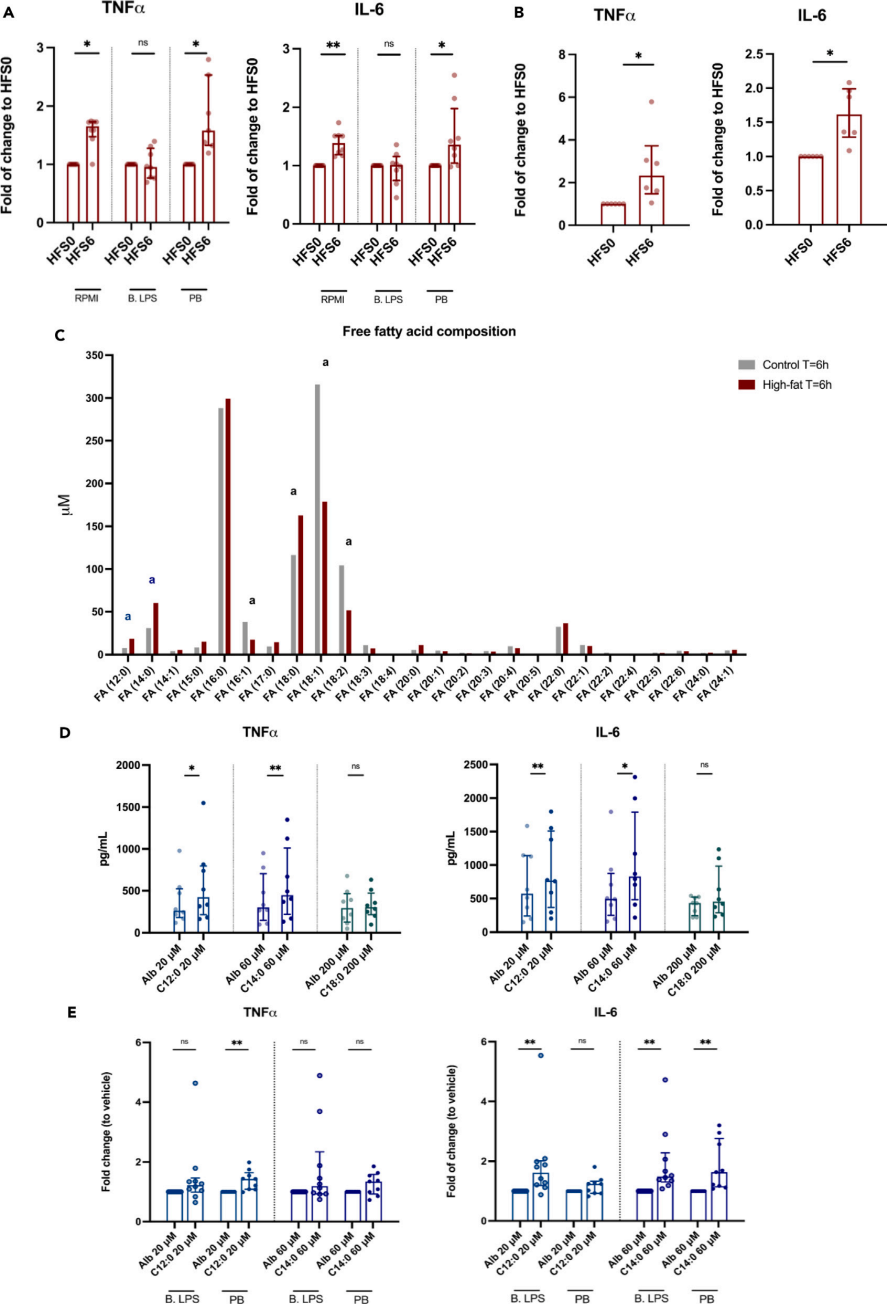
High-fat-shake-induced trained immunity is regulated via TLR4 but is not mediated via low-dose LPS, triglyceride-rich lipoproteins, or the saturated fatty acids that are particularly increased after the high-fat shake (A) Adherent human monocytes were pre-incubated for 1 h with plain RPMI or *Bartonella* LPS (B. LPS). After pre-incubation, the cells were exposed for 24 h to high-fat serum obtained at t = 0 h (HFS0) or at t = 6 h (HFS6), with (PB) or without neutralization of LPS. On day 6, cells were restimulated for 24 h and cytokine production was measured (*n* = 8). Data are presented as fold of change to HFS0 for each separate inhibitor. (B) HFS0 and HFS6 were depleted from apoB-containing lipoproteins before use in the training experiments (*n* = 6). (C) The fatty acid composition of the pooled serum obtained at t = 6 h after consumption of the high-fat and the reference shake. Lauric acid (FA(12:0))^a^, myristic acid (FA(14:0))^a^, and stearic acid (FA(18:0))^a^ were particularly higher in the high-fat serum. Palmitoleic acid (FA(16:1))^a^, oleic acid (FA(18:1))^a^, and linoleic acid (FA(18:2))^a^ were lower. (D) Monocytes were stimulated for 24 h with albumin-conjugated C12:0, C14:0, and C18:0 or with the albumin vehicle (Alb) alone. After resting and differentiation, cytokine production upon restimulation was measured (*n* = 8). (E) The same inhibition experiments as described under (A) were performed for C12:0 and C14:0. Data are presented as fold of change to the vehicle control for each separate inhibitor (B. LPS *n* = 10, PB *n* = 9). Median ± IQR. ∗ indicates two-sided *p* < 0.05, ∗∗*p* < 0.01, Wilcoxon signed-rank test. See also Figures S3 and S4.

Next, we aimed to identify which components of the post-high-fat serum induced the trained immunity response *in vitro*. Given the observation that cytokine production increases in a dose-response-like trend with the strongest response after stimulation with the serum obtained at t = 6 h, we investigated some specific serum components at this time point in more detail. Since low-dose LPS is an established inducer of trained immunity^18^^,^^19^ and a high-fat challenge has been shown to induce a transient increase in blood LPS levels (metabolic endotoxemia),^7^^,^^8^ we first focused on the contribution of LPS, which activates TLR4. Pre-incubation of the adherent monocytes with the specific TLR4 antagonist *Bartonella* LPS (B. LPS) before addition of the serum abolished the increased production of TNF-α and IL-6 after restimulation with LPS (Figure 3A), and also the IL-6 production, but not TNF-α production, after P3C restimulation (Figure S3A). Pre-treatment of the pooled serum with the LPS neutralizing Polymyxin B (PB) did not reduce cytokine production capacity (Figures 3A and S3A). Alongside these experiments, we also performed a control experiment to ensure effective blockage of TLR4 by B. LPS and effective neutralization of LPS by PB. In this control experiment, we stimulated the same adherent monocytes for 24 h with the TLR4 agonist *E. Coli* LPS with or without co-incubation of B. LPS and PB. After 24 h, cytokine production was assessed and showed an effective inhibition via B. LPS and PB (Figure S4A). Moreover, persistent blockage of TLR4 by B. LPS on day 6 was excluded by the observation that there is no difference in LPS-induced cytokine production between the RPMI controls with and without the co-incubation of B. LPS during the first 24 h of the training protocol (Figure S4B). Altogether, this indicates that the serum-induced activation of trained immunity occurs via TLR4 but is not solely mediated by metabolic endotoxemia.

Secondly, we assessed the specific role of TRLs. We performed the same set of pooled serum experiments as described earlier, but after serum depletion of apolipoprotein B (apoB)-containing lipoproteins, including the TRLs chylomicrons and very low-density lipoprotein. Lipid depletion of the serum did not abolish the trained immunity effect of the pooled post-high-fat-challenge serum (Figures 3B and S3B).

Lastly, the involvement of fatty acids (FAs) was assessed, which have been shown to affect innate immune cell homeostasis and have previously been described as potential TLR4 agonists.^20^ To this end, we compared the FFA composition in the pooled sera from the high-fat and reference shake at t = 6 h: higher concentrations of saturated FAs (SFAs) lauric acid (C12:0), myristic acid (C14:0), and stearic acid (C18:0) and lower concentrations of unsaturated FAs palmitoleic acid (C16:1), oleic acid (C18:1), and linoleic acid (C18:2) were found in the pooled sera obtained after the high-fat shake (Figure 3C). Stimulation of adherent monocytes with C12:0 and C14:0 indeed induced trained immunity, but only upon secondary stimulation with LPS and not with P3C (Figures 3D and S3C). For C18:0 we did not see an increase in cytokine production capacity. While the pre-incubation of monocytes with B. LPS impedes the induction of trained immunity after exposure to pooled serum, it did not completely inhibit cytokine production capacity after stimulation with C12:0 or C14:0 (Figures 3E and S3D).

### Immune cell distributions, innate immune phenotype, and cytokine production capacity were not altered after a single high-fat challenge

**Figure 4:**
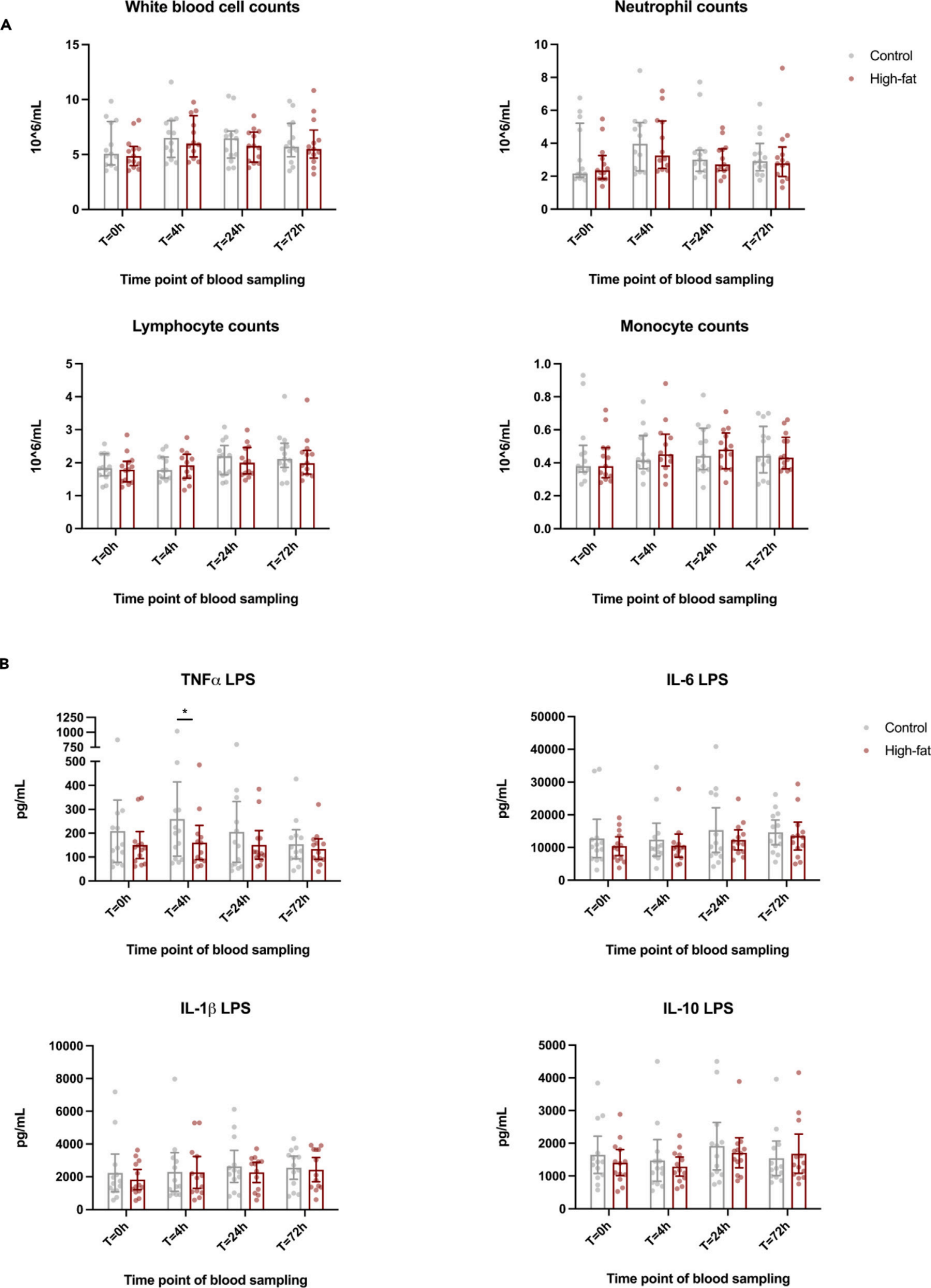
A single high-fat challenge does not induce persistent changes in white blood cell composition and does not induce increased cytokine production capacity in circulating PBMCs in healthy human volunteers (A) Total white blood cell counts, neutrophil counts, lymphocyte counts, and monocyte counts before and at several time points (t = 0 h, t = 4 h, t = 24 h, and t = 72 h) after the consumption of the high-fat and reference shake (*n* = 13). (B) *Ex vivo* cytokine production capacity of PBMCs isolated from the study participants at the same time points (*n* = 13). There was no significant difference in the PBMC TNF-α production upon LPS restimulation at t = 72 h, as calculated with the Wilcoxon signed-rank test (one-sided *p* = 0.31). Except for a lower PBMC TNF-α production upon LPS stimulation after the high-fat shake compared to the reference shake at t = 4 h, there were no significant changes, as calculated with linear mixed model for repeated measures. Median ± IQR. ∗ indicates *p* < 0.05, and no sign indicates no significance.

To investigate whether a single high-fat challenge can also induce persistent pro-inflammatory effects *in vivo*, we assessed the innate immune cell composition, activation, and cytokine production capacity after consumption of the high-fat shake compared to the reference shake at a long-term time point of 3 days (t = 72 h), next to time points t = 4 h and t = 24 h. For both the long-term and short-term time points, there were no alterations in white blood cell composition (Figure 4A) or monocyte subpopulations, i.e., the classical, intermediate, and non-classical monocytes (Table 2). Similarly, HLA-DR, CCR2, and CD11b expression on monocytes was not altered (Table 2). Moreover, we did not observe any significant changes in cytokine production capacity of peripheral blood mononuclear cells (PBMCs) at the long-term time point (Figure 4B). At t = 4 h, PBMC production of TNF-α upon *ex vivo* stimulation with LPS was significantly lower (*p* = 0.0195) after the high-fat shake compared to the reference shake. There were no significant differences in effects of time, shake, and shake × time interaction, nor was a carryover effect observed as analyzed by the linear mixed model.

**Table 2:** Immunophenotype of circulating monocytes

Monocyte immunophenotype	Shake type	Time point t = 0 h	t = 4 h	t = 24 h	t = 72 h	*p* value shake	Time	Shake × time
Classical monocytes, % gated of total mono’s	Ref HF	83.1 [79.9–86.3] 84.1 [80.9–87.3]	80.0 [76.8–83.2] 80.7 [77.5–83.2]	81.8 [78.6–85.0] 83.5 [80.3–86.7]	80.9 [77.6–84.1] 81.2 [78.0–84.4]	Ns	Ns	Ns
Intermediate monocytes, % gated of total mono’s	Ref HF	7.0 [5.5–8.5] 6.5 [5.0–8.0]	8.0 [6.5–9.5] 8.0 [6.6–9.5]	7.3 [5.9–8.8] 7.1 [5.7–8.6]	6.8 [5.4–8.3] 7.6 [6.2–9.1]	Ns	Ns	Ns
Non-classical monocytes, % gated of total mono’s	Ref HF	9.9 [7.3–12.5] 9.4 [6.8–12.0]	12.0 [9.4–14.6] 11.3 [8.7–13.9]	10.9 [8.3–13.4] 9.4 [6.8–12.0]	12.3 [9.7–14.9] 11.2 [8.6–13.8]	Ns	Ns	Ns
CCR2+ monocytes, % gated of total mono’s	Ref Hf	90.7 [88.7–92.7] 90.9 [88.8–92.9]	89.8 [87.8–91.8] 91.2 [89.2–93.2]	90.6 [88.6–92.6] 91.8 [89.8–93.8]	89.5 [87.5–91.5] 89.9 [88.0–91.3]	Ns	Ns	Ns
CD11b+ monocytes, % gated of total mono’s	Ref Hf	95.4 [92.2–98.7] 96.5 [93.1–99.9]	97.0 [93.7–100.0] 98.5 [95.3–100.0]	98.3 [95.1–100.0] 98.2 [95.0–100.0]	95.7 [92.5–99] 96.6 [93.4–99.8]	Ns	Ns	Ns
CCR2+ monocytes, MFI	Ref HF	3543 [3070–4017] 3507 [3023–3991]	3688 [3215–4162] 3813 [3340–4286]	4029 [3556–4502] 3974 [3501–4447]	3714 [3241–4188] 3467 [2994–3941]	Ns	Ns	Ns
CD11b+ monocytes, MFI	Ref Hf	11611 [8598–14624] 9883 [6783–12982]	11416 [8403–14429] 9993 [6980–13005]	9724 [6711–12738] 9396 [6384–12409]	8718 [5704–11731] 8821 [5809–11834]	Ns	Ns	Ns
HLA-DR monocytes, MFI	Ref Hf	16660 [14322–18998] 16391 [14009–18773]	17816 [15478–20154] 17357 [15020–19695]	17437 [15099–19775] 16578 [14241–18916]	15139 [12801–17477] 16228 [13890–18565]	Ns	Ns	Ns

Circulating monocyte subsets and expression of activation markers on total monocytes (*n* = 13). Data are missing for one participant. Missing data were fitted with the restricted maximum likelihood approach. Estimated marginal means with 95% confidence interval, as calculated with linear mixed model for repeated measures. Ref, reference shake; HF, high-fat shake; MFI, median fluorescence intensity. See also Figure S6.

### The duration of exposure to postprandial serum determines the trained immunity response

We proposed that the discrepancy between the *ex vivo* results and *in vivo* results is explained by the shorter duration of exposure to post-prandial metabolites *in vivo*. Therefore, we explored whether trained immunity could also be induced after a shorter incubation period with postprandial serum that better resembles the duration of metabolite changes *in vivo* after consumption of a high-fat shake. Exposure for 2 h instead of 24 h did not evoke an increased cytokine response (Figure S5).

## Discussion

This study aimed to test the hypothesis that transient dietary high-fat challenges trigger a prolonged activation of circulating innate immune cells, called trained immunity, which could promote atherosclerosis. A single high-fat challenge rapidly induced changes in the serum composition with the potential to induce trained immunity in healthy donor monocytes *in vitro*. However, this did not translate into a (persistent) hyperinflammatory monocyte phenotype *in vivo*.

Given the observation that post-high-fat-challenge serum induces *trained immunity in vitro* in healthy human monocytes, we considered several potential mediators of this effect. First, we assessed the contribution of metabolic endotoxemia. In this regard, we neutralized LPS from the serum with PB or antagonized its receptor TLR4 using B. LPS. We demonstrated that the increased monocyte cytokine production capacity could be abolished by blocking of TLR4 signaling but not by neutralization of endogenous LPS, thereby ruling out low-dose LPS as a possible mediator. Interestingly, B. LPS did not prevent the post-high-fat-shake-serum-induced increased production of TNF-α after P3C restimulation, suggesting that there might be additional, TLR4-independent pathways that also contribute to the trained immunity effect.

We previously showed that oxidized LDL (but not unmodified LDL) and lipoprotein(a)^12^^,^^13^ are potent inducers of trained immunity *in vitro*. Since increased blood TRL levels are causally associated with inflammation and atherosclerotic CVD,^2^ we subsequently explored the direct contribution of postprandial TRLs. Depletion of apoB-containing lipoproteins from the post-high-fat-challenge serum, however, did not abolish the increased cytokine production capacity upon secondary stimulation. Also, in previous studies it was shown that monocyte activation was enhanced after stimulation with postprandial blood TRLs, but only after treatment with lipoprotein lipase.^21^^,^^22^ This suggests a more specific role for the FAs, either contained in the TRLs or circulating as FFAs in the bloodstream.

Using a lipidomic platform we identified higher concentrations of several SFAs, including C12:0, C14:0, and C18:0, in the pooled serum obtained after the high-fat shake compared to the reference shake. Stimulation of healthy human monocytes with C12:0 and C14:0 indeed induced augmented cytokine production capacity upon secondary stimulation. The capability of these SFAs to induce trained immunity was also suggested in a recent study by Seufert et al.^23^ They showed that bone marrow-derived macrophages (BMDMs) and monocytes (BM monocytes) from wild-type mice, which were fed an SFA-enriched ketogenic diet for 2 weeks, were not inherently hyperinflammatory but only exhibited increased production of inflammatory cytokines upon stimulation with LPS, indicative of a trained immune phenotype. The potential of C18:0 to induce a persistent hyperresponsive state via TLR4 signaling in wild-type mice BMDM has also previously been demonstrated.^24^ However, in our studies in human monocytes we did not see a persistent induction of cytokine production upon training with C18:0. Apart from the differences in species, a possible explanation for the difference in these findings might be that we applied the first stimulation with C18:0 to monocytes, which were then differentiated into macrophages, while in the mice study BM cells were first differentiated into mature macrophages before applying the first stimulation with C18:0.

Notably, SFAs have previously been described as potential agonists of TLR4.^20^ In line with this, TLR4 deficiency protects rodents against FFA-induced inflammation and atherosclerosis upon feeding of a high SFA diet.^25^^,^^26^^,^^27^ In our experiments, primary stimulation of healthy human monocytes with SFAs induced augmented cytokine production capacity upon secondary stimulation; however, this could not be inhibited by antagonizing TLR4. Recent evidence also argues against a direct function of TLR4 as an FA receptor.^28^ Instead, it was proposed that SFAs provide a second hit of activation that is dependent upon prior TLR4 activation, potentially via metabolic endotoxemia.^28^ In summary, these results indicate that the persistent inflammatory changes in primary human monocytes after exposure to post-high-fat-challenge serum occur via TLR4 signaling but are not merely effectuated by TRLs, SFAs, or metabolic endotoxemia.

In the same cohort of healthy human volunteers from which we obtained the postprandial serum, the single high-fat challenge did not induce significant long-term immunological effects *in vivo*. At t = 24 h and t = 72 h we did not observe any persistent pro-inflammatory changes in *ex vivo* PBMC cytokine production capacity, white blood cell composition, or alterations in activation markers on monocytes. Also at the short-term time point (t = 4 h), we did not find strong alterations in innate immune cell activation (except for a lower TNF-α production upon stimulation with LPS), even though previous high-fat-challenge studies in healthy human volunteers have shown increased activation markers and cytokine response in circulating monocytes during the acute postprandial phase.^3^^,^^4^ The apparent discrepancy between our *in vitro* and *in vivo* findings are likely to be explained by the different time periods during which the cells were exposed to the postprandial changes. In our *in vivo* study, high concentrations of postprandial plasma metabolites are only present in the blood for a few hours, before returning back to the baseline. In the *in vitro* experiments, however, the monocytes were exposed to postprandial serum obtained at t = 6 h for a period of 24 h. To test the hypothesis that the induction of *trained immunity in vivo* is absent due to limited time exposure to postprandial metabolites, we exposed healthy human monocytes to postprandial serum for only 2 h, instead of 24 h. Indeed, in the case of short exposure to the postprandial serum, we did not induce significant changes in cytokine production capacity upon secondary stimulation. This suggests that one single high-fat challenge might not have the capacity to induce a persistent inflammatory response *in vivo* but that it requires a more prolonged exposure as occurs in the situation of a daily high-fat diet. Another important difference between the *in vitro* and *in vivo* situation is that persistent reprogramming of monocytes *in vivo* is dependent on hematopoietic stem and progenitor cell (HSPC) reprogramming,^29^ as the half-life of circulating monocytes is only about one day.^30^ It could be that the HSPCs require a stronger or more prolonged stimulus to mount *trained immunity* than mature monocytes. Notably, in an *apoE*^−/−^ mouse model of atherosclerosis, a 4-week WTD induced profound inflammatory reprogramming of circulating innate immune cells and their bone marrow progenitors, which persisted despite switching back to regular diet.^17^ Activation of the NLRP3 inflammasome and IL-1β signaling played a fundamental role in the progenitor reprogramming, inducing long-term epigenetic modifications and a myeloid-biased differentiation. Also, in primates it was shown that high-fat diet feeding resulted in increased inflammation, monocyte activity, and more histone H3 lysine 27 (H3K27) acetylation,^31^ an epigenetic mark previously described to play a role in trained immunity.^32^

### Limitations of the study

Our study has several strengths and limitations. First, our study population consisted of young healthy lean individuals, and our findings might have been different in patients with obesity and metabolic syndrome. Secondly, the variation in biological processes occurring after the consumption of a meal is large, and it is hard to identify the individual contribution of specific underlying mechanisms *in vivo*. Additionally, since previous *in vitro* studies have shown that a trained immune phenotype has already been developed 3 days after removal of the primary stimulus,^26^ we selected the PBMC cytokine production capacity at the postprandial time point T = 72 h as our main endpoint. In this way, possible encounters with other immunomodulating factors during the period of *trained immunity* development in the *in vivo* setting have been minimized as much as possible. Third, although we showed that high-fat-serum induced *trained immunity* in terms of persistent augmented cytokine production capacity, we did not perform additional analyses on the underlying epigenetic and metabolic processes that characterize *trained immunity*. Lastly, due to the small sample size, no analysis on sex differences could be performed.

An important strength of our study is the robust cross-over experimental design with the careful control for confounders such as diet, exercise, and circadian rhythm. Many previous postprandial studies with high-fat challenges mostly used fasting or water as a reference. However, in these studies it could not be ruled out that the observed effects are also evoked by a common meal. In order to distinguish between a high-fat challenge and a common meal response, we used an averagely consumed breakfast shake as a reference. As a consequence, both shakes were not isocaloric and the high-fat challenge contained less carbohydrates at the expense of the increased fat content compared to the reference shake. Our *in vitro* experiments with postprandial serum showed that the reference shake did not induce a pro-inflammatory response in healthy human monocytes, indicating that the effects found after the high-fat-challenge serum do not generally occur after the consumption of a common meal.

In conclusion, while the short-term exposure to a single high-fat challenge is not sufficient to induce significant immunological effects *in vivo*, it does lead to alterations in the serum composition that have the potential to induce persistent innate immune cell activation in case of longer exposure. Future studies on the connection between innate immune cell reprogramming and WTD in humans could allow for the identification of additional therapeutic opportunities to lower the susceptibility for cardiovascular events and other chronic inflammatory diseases.

## Resource availability

### Lead contact

Further information and requests for resources and reagents should be directed to and will be fulfilled by the lead contact, dr. Niels Riksen (Niels.Riksen@radboudumc.nl).

### Materials availability

This study did not generate new unique reagents.

### Data and code availability

• Data reported in this paper will be shared by the lead contact upon request.
• Code: this study did not develop new codes.
• Other times: not applicable.

## Acknowledgments

We would like to thank Marlies Noz and Cor Jacobs for their help with the flow cytometry experiments and the research nurses of the Radboud Technology Center for Clinical Studies for their help with the blood drawings on study days. M.G.N., L.A.B.J., and N.P.R. were supported by a grant from the Dutch CardioVascular Alliance/Dutch Heart Foundation (CVON2018-27). N.P.R. was further supported by a grant of the ERA-CVD Joint Transnational Call 2018, which is supported by the Dutch Heart Foundation in The Hague (JTC2018, project MEMORY; 2018T093). M.G.N. is further supported by an ERC Advanced Grant (FP/2007-2013/ERC grant 2012-322698), and a Spinoza Prize (NWO SPI 92-266). S.B. is supported by the Dutch Heart Foundation (2018T028).

## Author contributions

J.v.T.: conceptualization, methodology, validation, formal analysis, investigation, resources, data curation, writing – original draft, visualization, and project administration. J.I.P.v.H.: investigation and writing – review and editing. H.B.: investigation and writing – review and editing. J.W.: investigation and writing – review and editing. Y.M.t.H.: investigation and writing – review and editing. M.G.: investigation and writing – review and editing. H.Z.-v.E.: methodology and writing – review and editing. L.R.: software, writing – review and editing, formal analysis, and data curation. L.A.B.J.: writing – review and editing, supervision, and funding acquisition. M.G.N.: writing – review and editing, supervision, and funding acquisition. L.A.A.: conceptualization, methodology, and writing – review and editing. S.B.: conceptualization, methodology, investigation, formal analysis, writing – review and editing, supervision, and funding acquisition. N.P.R.: conceptualization, methodology, investigation, resources, and writing – review and editing, supervision, project administration, and funding acquisition.

## Declaration of interests

M.G.N. and L.A.B.J. are scientific founders of TTxD and Lemba Therapeutics.

## STAR★Methods

### Key resources table

**Table:** 

REAGENT or RESOURCE	SOURCE	IDENTIFIER
**Antibodies**		

Anti-human CD45 KromeOrange, Clone J33	Beckman Coulter	Cat# A96416; RRID: AB_2888654
Anti-human HLA-DR PE, Clone immu-357	Beckman Coulter	Cat# IM1638U; RRID: AB_2876782
Anti-human CD14 PECy7, Clone 61D3	eBioscience	Cat# 25-0149-42; RRID: AB_1582276
Anti-human CD16 FITC, Clone CD16	eBioscience	Cat#: 11-0168-42; RRID: AB_10805747
Anti-human CD3 APC-Alexa750, Clone UCTH1	Beckman Coulter	Cat# A66329; RRID: AB_2876783
Anti-human CD56 APC, Clone N901	Beckman Coulter	Cat# IM2474U; RRID: AB_2876784
Anti-human CD192 (CCR2) BV421, Clone 48607	Becton Dickinson	Cat# 564067; RRID: AB_2738573
Anti-human CD11b BV785, Clone ICRF44	Biolegend	Cat# 301346; RRID: AB_2563794
Anti-human CD36 PC5.5, Clone 5-271	Biolegend	Cat# 336202; RRID: AB_1279228

**Biological samples**		

Peripheral blood through venous puncture, 18–40 years old males and females	Homo Sapiens	Medical Ethics Committee of the Region Arnhem-Nijmegen ethical approval number N167894.091.18
Buffy coat	Homo Sapiens	Sanquin Blood Bank, Nijmegen, The Netherlands

**Chemicals, peptides, and recombinant proteins**		

Pharm Lyse lysing buffer	BD Biosciences	Cat# 555899
Roswell Park Memorial Institute (RPMI) 1640 Dutch-modified culture medium	Life Technologies/Invitrogen	Cat# 22409015
Glutamine, 2 mmol/L in RPMI	Invitrogen	Cat# 25030081
Gentamycin, 10 mg/mL in RPMI	Centrafarm, Etten Leur, The Netherlands	
Pyruvate, 1 mM in RPMI	Invitrogen	Cat# 11360070
Lipopolysaccharide from *Escherichia coli*	Sigma-Aldrich, Serotype 055:B5	Cat# L2880
Pam3CysK4	EMC Microcollections	Cat# L2000
β1,3-(D)glucan (β-glucan)	Saeed et al.^33^	N/A
Bacille Calmette-Guérin Vaccine	Statens Serum Institute	N/A
Bartonella quintana CIP 103739 strain	Hirschfel et al.^34^	N/A
Polymyxin B	Sigma-Aldrich	Cat# 1405-20-5
Lauric acid	Sigma-Aldrich	Cat# 143-07-7
Myristic acid	Sigma-Aldrich	Cat# 544-63-8
Stearic acid	Sigma-Aldrich	Cat# 57-11-4
Bovine serum albumin solution	Sigma-Aldrich	Cat# 9048-46-8
Ficoll-Paque PLUS density gradient media	Cytiva Life Sciences	Cat# 17144003
Percoll	Sigma-Aldrich	Cat# P1644
Phosphate buffered saline (1×) without calcium and magnesium	Lonza	Cat# BE17-516F
Polyethylene glycol 8000	Sigma-Aldrich	Cat# 25322-68-3
Bartonella quintana CIP 103739 strain	Hirschfel et al.^34^	N/A

**Critical commercial assays**		

Human Duoset IL-6 ELISA	R&Dsystems	Cat# DY206; RRID: AB_2814717
Human Duoset TNF ELISA	R&Dsystems	Cat# DY210; RRID: AB_2848160
Human Duoset IL-10 ELISA	R&Dsystems	Cat# DY217B; RRID: AB_2927688
Human Duoset IL-1b ELISA	R&Dsystems	Cat# DY201; RRID: AB_2848158

**Software and algorithms**		

Kaluza	Beckman Coulter	Version 2.1
Graphpad Prism	Graphpad software	Version 9.3.0
Lipidomics Workflow Manager Software	Sciex	Version 1
Stata	StataCorp LLC	Version 17
SPSS version 25	SPSS Statistics, IBM Corp	Version 25

**Other**		

CytoFLEX flow cytometer	Beckman Coulter	N/A
Lipidyzer platform	Sciex	N/A
Nexera X2 LC system	Shimadzu	N/A
Sysmex-XN 450 hematology analyzer	Sysmex	N/A
Round-bottom 96-well plate	Corning	Cat# CLS351177
Flat-bottom 96-well plate	Corning	Cat# CLS351172

### Experimental model and study participant details

#### Ethical committee approval

Local approval was granted by the Medical Ethics Committee of the Region Arnhem-Nijmegen (ethical approval number N167894.091.18) and all experiments were in accordance with the Declaration of Helsinki. All subjects provided written informed consent. The trial is registered in ClinicalTrials.gov under NCT05682456. The study was conducted from May 2019 to December 2021 at the department of Internal Medicine, Radboudumc, the Netherlands.

#### Subjects

Healthy volunteers, aged between 18 and 40 years, were recruited. Exclusion criteria consisted of a BMI <18 or >27 kg/m^2^, LDL cholesterol levels >3.5 mmol/L and triglyceride levels >2 mmol/L, a history of previous cardiovascular events or any long-term medical condition. Additional exclusion criteria were the use of medication (with the exception of oral contraceptives) or supplements, smoking within the year before study entry, current infection or clinically significant infection within 1 month before study entry, vaccination within 3 months prior to study entry, abuse of drugs or alcohol, and a vegetarian or dairy free diet. More detailed information on the sample size can be found under the section “quantification and statistical analysis”. The baseline characteristics (i.e., age, sex and race) can be found in Table 1.

For the *in vitro* experiments, we obtained buffy coats from healthy donors via Sanquin Blood Bank, Nijmegen, The Netherlands, after written informed consent. Since the buffy coats where donated anonymously to us via Sanquin, we do not have additional information on age, sex and race.

### Method details

#### Study design

In a prospective randomized open label with blinded endpoint (PROBE) controlled cross-over design (Figure 1A), the participants were allocated by simple randomization to either start with the high-fat shake (high-fat challenge) or a reference shake (control shake) in a 1:1 ratio (see below for details on the shake content). Treatment allocation was not blinded to participants nor to the study team members, compatible with the PROBE design. Instead, outcome assessment was blinded for treatment allocation. The rationale for the cross-over design is based on the known considerable interindividual variation in cytokine production capacity and in trained immunity development.^35^ A design in which each participant serves as its own control, i.e., a cross-over design, therefore strongly increases power. To further reduce potential confounders, participants were randomized to start with the high-fat shake or control shake. To reduce the risk of any carry over effect, we included a wash-out period of one months, which we considered the optimum between reducing the risk of carry-over without increasing too much the risk for intercurrent confounders such as infections or vaccinations.

As part of the cross-over design, all patients received two shakes. Half of the participants started with the high-fat shake, the other half with the reference shake. After a wash-out period of at least 1 month, which was included to prevent any carry over effects, they received the alternative shake. Blood was drawn before (t = 0 h) and at several time points after the consumption of both shakes (t = 1 h, t = 2 h, t = 4 h, t = 6 h, t = 24 h, t = 72 h). Starting from the evening before the ingestion of the shake until time point t = 72 h, the participants followed standardized meal plans which were designed according to the Dutch food-based dietary guidelines.^36^ An example of a standardized meal plan is provided in the supplements (Table S1). The meal plans were adjusted to the subject’s personal caloric requirements as calculated with the WHO formula.^37^ The participants had to adhere to the exact same meal plans during both experimental periods. Before the blood drawing at t = 0 h, t = 24 h and t = 72 h participants were asked to have an overnight fast for 10–14 h. Additionally, they had to refrain from alcohol and strenuous exercise the day before and during study days. The means of transportation to the research location were similar for each study day. Also, the timing of shake consumption and sample collection did not differ between the high-fat and reference shake to avoid any potential interference of circadian rhythms.^38^

#### Shakes

The high-fat shake containing 95g of fat consisted of 53% (w/v) fresh cream, 3% (w/v) sugar and 44% (w/v) water. The reference shake contained the same nutritional and energetic value as an average breakfast as previously described by Esser et al.^5^ This reference shake containing 14.5 g of fat consisted of 43% (w/v) full cream milk, 48% (w/v) full cream yoghurt, 4% (w/v) lemonade, 4% (w/v) fantomalt (Nutricia B.V., the Netherlands) and 1% (w/v) wheat fiber. The nutritional values are listed in Table S2. The reference shake was used to acquire a common postprandial response and the shakes were therefore not isocaloric. Both shakes had a total volume of 500 mL and had to be consumed within 10 min.

#### Cardiovascular and anthropometric assessment for baseline characterization

For the baseline characterization, information on medical history, smoking status and medication use were obtained from all participants. The participants were also asked to keep a food diary for three days in order to get an overview of the habitual diet of the participants. Blood pressure and heart rate were measured with a manual sphygmomanometer in supine position after at least 10 min of rest.

Creatinine, glucose, total cholesterol (Tc), high-density lipoprotein cholesterol (HDLc) and triacylglycerol (TG) were measured by the hospital laboratory (Radboudumc, Nijmegen, the Netherlands) using standardized methods, and low-density lipoprotein cholesterol (LDLc) was calculated with the Friedewald formula.

#### Blood sampling during study days

Plasma TG, insulin, glucose and serum free fatty acid (FFA) concentrations were immediately assessed before (t = 0 h) and at t = 1 h, 2 h, 4 h, and 6 h after shake consumption by the hospital laboratory (Radboudumc, Nijmegen, the Netherlands) according to standardized methods.

At t = 0 h, t = 2 h, t = 4 h and t = 6 h a second set of serum blood samples were centrifuged for 10 min at 3800 rpm at 4°C after allowing the blood to clot for at least 30 min. The serum was removed under sterile conditions and stored at −80°C. After study completion of all 14 study participants, the sera from each individual (with the exception of one participant since signs of an infection were observed) were thawed on ice and pooled for each separate time point for the reference-as well as the high-fat shake and stored again at −80°C until further use in *in vitro* experiments. Acquisition and quantification of specific FFAs in pooled serum was performed by using the Lipidyzer platform consisting of a Qtrap 5500 mass spectrometer (Sciex) with DMS, coupled to a Shimadzu Nexera X2 LC system for flow injection, and analyzed by the Lipidomics workflow manager software. A further detailed description of the FFA quantification methods can be found in references.^39^

Before (t = 0 h) and at t = 4 h, t = 24 h and t = 72 h after consumption of both shakes, blood was collected in EDTA vacutainers for subsequent peripheral blood mononuclear cell (PBMC) isolation. Total blood cell counts and leukocyte differentiation were determined with an automated Sysmex-XN 450 hematology analyzer (Sysmex).

#### PBMC isolation and stimulation

PBMCs were isolated by dilution of blood in pyrogen-free phosphate buffered saline (PBS) (Lonza) and subsequent Ficoll-Paque density gradient centrifugation (Cytiva Life Sciences). The PBMCs were washed thrice in PBS and eventually resuspended in Roswell Park Memorial Institute 1640 Dutch-modified culture medium (RPMI) (Life Technologies/Invitrogen) supplemented with 2 mmol/L glutamine (Invitrogen), 50 μg/mL gentamicin (Centrafarm) and 1 mmol/L pyruvate (Invitrogen). The cell counts and composition of the PBMC fraction were assessed by the automated Sysmex analyzer. To evaluate cytokine production capacity, 5 × 10^5^ PBMCs per well were stimulated for 24 h in round-bottom 96-well plates (Corning) with RPMI or with 10 ng/mL lipopolysaccharide (LPS) purified from Escherichia coli (Sigma-Aldrich, Serotype 055:B5) via phenol re-extraction.^34^ After 24 h, the plates were centrifuged and supernatants were stored at −80°C until cytokine measurement.

#### Cytokine concentration measurements

Cytokine production was determined in supernatants using commercially available DuoSet ELISA kits (R&D Systems) for IL (interleukin)-6, Tumor necrosis factor- α (TNF-α), IL-1β and IL-10 according to the manufacturer’s instructions.

#### Flow cytometry

Monocyte subpopulations and expression markers were identified with flow cytometry. Using the lysis-no-wash strategy (BD Pharm Lyse lysing buffer, Becton Dickinson), 50 μL EDTA blood was stained with monoclonal antibodies targeting CD45 (anti-human CD45 KromeOrange, Clone J33, Beckman Coulter), CD16 (anti-human CD16 FITC, Clone CD16, eBioscience), CD14 (anti-human CD14 PECy7, Clone 61D3, eBioscience), CD3 (anti-human CD3 APC-Alexa750, clone UCTH1, Beckman Coulter), CD56 (anti-human CD56 APC, Clone N901, Beckman Coulter), HLA-DR (anti-human HLA-DR PE, Clone immu-357, Beckman Coulter), CD11b (anti-human CD11b BV785, Clone ICRF44, Biolegend), CCR2 (anti-human CD192 (CCR2) BV421, Clone 48607, Becton Dickinson) and CD36 (anti-human CD36 PC5.5, Clone 5–271, Biolegend). Subsequently, the cell populations and expression markers were measured with CytoFlex flow cytometer (Beckman Coulter), which underwent daily quality control to correct for variation in laser settings. Data were analyzed by manual gating with Kaluza 2.1 software (Beckman Coulter). Gates were set using the fluorescence-minus-one (FMO) method and compensation was performed using compensation beads. FMO analysis revealed that the CD36 antibody signal had significant overlap with autofluorescence and was therefore excluded from further analysis. Characterization of monocytes subsets was performed according to current recommendations.^40^^,^^41^ The applied gating strategy is shown in Figure S6.

#### Isolation of peripheral blood mononuclear cells and monocytes from buffy coat donors

PBMCs were isolated from buffy coats using Ficoll-paque as described above. We subsequently enriched the monocyte fraction with hyperosmotic Percoll gradient isolation (Sigma Aldrich). An extra purification step was achieved by adhering Percoll isolated monocytes to polysterene flat bottom plates (Corning) for 1 h at 37°C. To yield maximal purity, a subsequent washing step with PBS was performed.

#### Trained immunity experiments with postprandial pooled serum

For the experiments with the pooled sera obtained from the study participants we used our established *in vitro* model for trained immunity.^42^^,^^43^ In short, adherent monocytes from healthy donors (see above) were stimulated for either 2 h or 24 h with RPMI culture medium containing 10% of fasting serum (t = 0 h) and RPMI containing 10% of postprandial serum obtained at t = 2, 4 or 6 h after either the reference- or high-fat shake. RPMI containing 10% of human pooled serum was included in the experiments as a negative control and 2 μg/mL β-1,3-(D)-Glucan^33^ (β-glucan, kindly provided by professor David Williams, TN, USA) or 5 μg/mL Bacille Calmette-Guérin (BCG) vaccine (Statens Serum Institute) as an established positive control for *in vitro* trained immunity.^42^ Donors were excluded from the subsequent statistical analysis when the fold change of the positive control (β-glucan or BCG) compared to the negative control (RPMI) was lower than 1.5. After 24 h of stimulation with the serum, the cells were washed once with warm PBS and incubated for 5 days in RPMI with 10% human pooled serum. After the resting period, cells were stimulated for a second time during another 24 h with either RPMI, LPS 10 ng/mL (TLR4 agonist) or Pam3CysK4 (P3C) 10 μg/mL (L2000, EMC Microcollections; TLR2 agonist). The same set of experiments was also performed with pooled postprandial serum in which the apoB-containing lipoprotein fraction was precipitated with polyethylene glycol 8000 (Sigma Aldrich) and removed from the serum.^44^

In the inhibition experiments, cells were first pre-incubated for 1 h with 100 ng/mL *Bartonella* LPS (B. LPS; a potent TLR-4 antagonist), extracted from the *Bartonella quintana* CIP 103739 strain^34^ before exposure to the participant’s serum. For inhibition with Polymyxin B (PB) (Sigma Aldrich), the sera were pre-incubated with 2 μg/mL PB for 2 h before addition to the cells.

#### Myristic acid, lauric acid, and stearic acid training experiments

In a separate set of training experiments, adherent monocytes were stimulated with lauric acid (C12:0) (Sigma-Aldrich), myristic acid (C14:0) (Sigma-Aldrich) and stearic acid (C18:0) (Sigma-Aldricg). Stock C12:0, C14:0 and C18:0 were each dissolved in 100% ethanol. C12:0 and C14:0 were each conjugated to endotoxin-free albumin (Sigma-Aldrich) by warming to 37°C in a water bath before combining in a 1:5 ratio. C18:0 was warmed to 60°C before combining in a 1:5 ratio with albumin. The three separate mixtures were sonicated for 20–25 min and kept at 37°C until use. The vehicle control for 20 μM C12:0 consisted of 0.010% albumin and 0.010% ethanol, the vehicle control for 60 μM C14:0 consisted of 0.030% albumin and 0.030% ethanol and the vehicle control for 200 μM C18:0 consisted of 0.10% albumin and 0.10% ethanol. In the inhibition experiments with B. LPS and PB the same methods were used as described above.

### Quantification and statistical analysis

Data were analyzed with SPSS (version 25.0, SPSS Statistics, IBM Corp) and Stata (version 17.0, StataCorp LLC). Graphs were generated using GraphPad Prism (version 9.3.0; GraphPad Software).

Baseline characteristics are presented as mean with SD when normally distributed or as median with minimum and maximum values for non-normally distributed continuous variables, and as percentages for categorical data.

Because of the non-Gaussian distribution of most inflammatory parameters, *in vitro* experiments were presented as median with interquartile range and analyzed using a Wilcoxon signed-rank test: raw *p*-values are shown. A two-sided *p*-value of <0.05 was considered statistically significant. In case of missing data no imputation was performed.

For the *in vivo* study, a sample size calculation was performed for the primary endpoint, which was *ex vivo* PBMC TNFα production upon LPS restimulation at t = 72 h. We hypothesized that the high-fat shake augmented LPS-induced TNFα production at t = 72 h, indicative of trained immunity, compared to the reference shake. We have based our sample size calculation on a previous study in which we concluded that the LPS-induced TNFα-release was 121.0 pg/mL, SD 139.3 (mean SD, *N* = 20) in healthy subjects without atherosclerosis,^15^ which is comparable to the group of healthy volunteers that we included in this study. Also, we measured LPS-induced TNFα in patients with atherosclerosis, which was 242.1 pg/mL, SD 305.4.^15^ Therefore, we considered a difference of 121 a clinically significant difference. We conducted the following sample size calculation: The pooled standard deviation is 235. Assuming a 30% lower pooled standard deviation in our group due to the paired experimental design, we need 14 patients to detect the true difference between treatments of 121,1 units with a power of 80% and a type I error rate of 5% (one-sided). This was based on the assumption that the within-patient standard deviation of the cytokine count is 165. To account for drop-outs, we included 2 additional subjects.

For the primary outcome, statistical comparison was performed using a Wilcoxon signed-rank test. For all the other outcomes, statistical comparisons were performed by fitting linear mixed models for repeated measures, using ‘period’, ‘group’, ‘shake’, ‘time point’ and ‘shake’ by ‘time point’ interaction as fixed effects and subject as random effect, then estimating contrasts between the shake types at each time point. Missing data in the postprandial metabolite changes and flow cytometry analysis were accounted for with the restricted maximum likelihood approach. A one-sided *p*-value <0.05 was considered significant for the *ex vivo* PBMC TNFα cytokine production upon LPS stimulation at t = 72 h. For the other outcomes a two-sided *p*-value <0.05 was considered significant.

Since signs of an infection (i.e., increased white blood cell counts and increased levels of C-reactive protein at baseline) were observed in one of the participants after completion of the study, this participant was excluded from the statistical analyses of inflammatory outcomes and serum samples were not included in the pooled serum for further use in the *in vitro* experiments.

The number of cell donors or participants (n) for all presented parameters are provided in the figure legends, as well as the applied statistical tests.

### Additional resources

The trial is registered in ClinicalTrials.gov under NCT05682456.

## References

[1] GBD 2017 DALYs and HALE Collaborators (2018). Global, regional, and national disability-adjusted life-years (DALYs) for 359 diseases and injuries and healthy life expectancy (HALE) for 195 countries and territories, 1990-2017: a systematic analysis for the Global Burden of Disease Study 2017. Lancet.

[2] Nordestgaard B.G. (2016). Triglyceride-Rich Lipoproteins and Atherosclerotic Cardiovascular Disease: New Insights From Epidemiology, Genetics, and Biology. Circ. Res..

[3] Gower R.M., Wu H., Foster G.A., Devaraj S., Jialal I., Ballantyne C.M., Knowlton A.A., Simon S.I. (2011). CD11c/CD18 expression is upregulated on blood monocytes during hypertriglyceridemia and enhances adhesion to vascular cell adhesion molecule-1. Arterioscler. Thromb. Vasc. Biol..

[4] van Oostrom A.J.H.H.M., Rabelink T.J., Verseyden C., Sijmonsma T.P., Plokker H.W.M., De Jaegere P.P.T., Cabezas M.C. (2004). Activation of leukocytes by postprandial lipemia in healthy volunteers. Atherosclerosis.

[5] Esser D., Oosterink E., op 't Roodt J., Henry R.M.A., Stehouwer C.D.A., Müller M., Afman L.A. (2013). Vascular and inflammatory high fat meal responses in young healthy men; a discriminative role of IL-8 observed in a randomized trial. PLoS One.

[6] Lemay D.G., Huang S., Huang L., Alkan Z., Kirschke C., Burnett D.J., Wang Y.E., Hwang D.H. (2019). Temporal changes in postprandial blood transcriptomes reveal subject-specific pattern of expression of innate immunity genes after a high-fat meal. J. Nutr. Biochem..

[7] Piya M.K., Harte A.L., McTernan P.G. (2013). Metabolic endotoxaemia: is it more than just a gut feeling?. Curr. Opin. Lipidol..

[8] Lyte J.M., Gabler N.K., Hollis J.H. (2016). Postprandial serum endotoxin in healthy humans is modulated by dietary fat in a randomized, controlled, cross-over study. Lipids Health Dis..

[9] Kleinnijenhuis J., Quintin J., Preijers F., Joosten L.A.B., Ifrim D.C., Saeed S., Jacobs C., van Loenhout J., de Jong D., Stunnenberg H.G. (2012). Bacille Calmette-Guerin induces NOD2-dependent nonspecific protection from reinfection via epigenetic reprogramming of monocytes. Proc. Natl. Acad. Sci. USA.

[10] Quintin J., Saeed S., Martens J.H.A., Giamarellos-Bourboulis E.J., Ifrim D.C., Logie C., Jacobs L., Jansen T., Kullberg B.J., Wijmenga C. (2012). Candida albicans infection affords protection against reinfection via functional reprogramming of monocytes. Cell Host Microbe.

[11] Kleinnijenhuis J., Quintin J., Preijers F., Joosten L.A.B., Jacobs C., Xavier R.J., van der Meer J.W.M., van Crevel R., Netea M.G. (2014). BCG-induced trained immunity in NK cells: Role for non-specific protection to infection. Clin. Immunol..

[12] Bekkering S., Quintin J., Joosten L.A.B., van der Meer J.W.M., Netea M.G., Riksen N.P. (2014). Oxidized low-density lipoprotein induces long-term proinflammatory cytokine production and foam cell formation via epigenetic reprogramming of monocytes. Arterioscler. Thromb. Vasc. Biol..

[13] van der Valk F.M., Bekkering S., Kroon J., Yeang C., Van den Bossche J., van Buul J.D., Ravandi A., Nederveen A.J., Verberne H.J., Scipione C. (2016). Oxidized Phospholipids on Lipoprotein(a) Elicit Arterial Wall Inflammation and an Inflammatory Monocyte Response in Humans. Circulation.

[14] Dominguez-Andres J., Dos Santos J.C., Bekkering S., Mulder W.J.M., van der Meer J.W.M., Riksen N.P., Joosten L.A.B., Netea M.G. (2023). Trained immunity: adaptation within innate immune mechanisms. Physiol. Rev..

[15] Bekkering S., van den Munckhof I., Nielen T., Lamfers E., Dinarello C., Rutten J., de Graaf J., Joosten L.A.B., Netea M.G., Gomes M.E.R., Riksen N.P. (2016). Innate immune cell activation and epigenetic remodeling in symptomatic and asymptomatic atherosclerosis in humans in vivo. Atherosclerosis.

[16] Bekkering S., Stiekema L.C.A., Bernelot Moens S., Verweij S.L., Novakovic B., Prange K., Versloot M., Roeters van Lennep J.E., Stunnenberg H., de Winther M. (2019). Treatment with Statins Does Not Revert Trained Immunity in Patients with Familial Hypercholesterolemia. Cell Metab..

[17] Christ A., Gunther P., Lauterbach M.A.R., Duewell P., Biswas D., Pelka K., Scholz C.J., Oosting M., Haendler K., Bassler K. (2018). Western Diet Triggers NLRP3-Dependent Innate Immune Reprogramming. Cell.

[18] Geng S., Chen K., Yuan R., Peng L., Maitra U., Diao N., Chen C., Zhang Y., Hu Y., Qi C.F. (2016). The persistence of low-grade inflammatory monocytes contributes to aggravated atherosclerosis. Nat. Commun..

[19] Ifrim D.C., Quintin J., Joosten L.A.B., Jacobs C., Jansen T., Jacobs L., Gow N.A.R., Williams D.L., van der Meer J.W.M., Netea M.G. (2014). Trained immunity or tolerance: opposing functional programs induced in human monocytes after engagement of various pattern recognition receptors. Clin. Vaccine Immunol..

[20] Christ A., Lauterbach M., Latz E. (2019). Western Diet and the Immune System: An Inflammatory Connection. Immunity.

[21] Ono-Moore K.D., Snodgrass R.G., Huang S., Singh S., Freytag T.L., Burnett D.J., Bonnel E.L., Woodhouse L.R., Zunino S.J., Peerson J.M. (2016). Postprandial Inflammatory Responses and Free Fatty Acids in Plasma of Adults Who Consumed a Moderately High-Fat Breakfast with and without Blueberry Powder in a Randomized Placebo-Controlled Trial. J. Nutr..

[22] den Hartigh L.J., Connolly-Rohrbach J.E., Fore S., Huser T.R., Rutledge J.C. (2010). Fatty acids from very low-density lipoprotein lipolysis products induce lipid droplet accumulation in human monocytes. J. Immunol..

[23] Seufert A.L., Hickman J.W., Traxler S.K., Peterson R.M., Waugh T.A., Lashley S.J., Shulzhenko N., Napier R.J., Napier B.A. (2022). Enriched dietary saturated fatty acids induce trained immunity via ceramide production that enhances severity of endotoxemia and clearance of infection. Elife.

[24] Hata M., Andriessen E.M.M.A., Hata M., Diaz-Marin R., Fournier F., Crespo-Garcia S., Blot G., Juneau R., Pilon F., Dejda A. (2023). Past history of obesity triggers persistent epigenetic changes in innate immunity and exacerbates neuroinflammation. Science.

[25] Holland W.L., Bikman B.T., Wang L.P., Yuguang G., Sargent K.M., Bulchand S., Knotts T.A., Shui G., Clegg D.J., Wenk M.R. (2011). Lipid-induced insulin resistance mediated by the proinflammatory receptor TLR4 requires saturated fatty acid-induced ceramide biosynthesis in mice. J. Clin. Invest..

[26] Shi H., Kokoeva M.V., Inouye K., Tzameli I., Yin H., Flier J.S. (2006). TLR4 links innate immunity and fatty acid-induced insulin resistance. J. Clin. Invest..

[27] Ding Y., Subramanian S., Montes V.N., Goodspeed L., Wang S., Han C., Teresa A.S., Kim J., O'Brien K.D., Chait A. (2012). Toll-like receptor 4 deficiency decreases atherosclerosis but does not protect against inflammation in obese low-density lipoprotein receptor-deficient mice. Arterioscler. Thromb. Vasc. Biol..

[28] Lancaster G.I., Langley K.G., Berglund N.A., Kammoun H.L., Reibe S., Estevez E., Weir J., Mellett N.A., Pernes G., Conway J.R.W. (2018). Evidence that TLR4 Is Not a Receptor for Saturated Fatty Acids but Mediates Lipid-Induced Inflammation by Reprogramming Macrophage Metabolism. Cell Metab..

[29] Riksen N.P., Bekkering S., Mulder W.J.M., Netea M.G. (2023). Trained immunity in atherosclerotic cardiovascular disease. Nat. Rev. Cardiol..

[30] Patel A.A., Zhang Y., Fullerton J.N., Boelen L., Rongvaux A., Maini A.A., Bigley V., Flavell R.A., Gilroy D.W., Asquith B. (2017). The fate and lifespan of human monocyte subsets in steady state and systemic inflammation. J. Exp. Med..

[31] Short J.D., Tavakoli S., Nguyen H.N., Carrera A., Farnen C., Cox L.A., Asmis R. (2017). Dyslipidemic Diet-Induced Monocyte "Priming" and Dysfunction in Non-Human Primates Is Triggered by Elevated Plasma Cholesterol and Accompanied by Altered Histone Acetylation. Front. Immunol..

[32] Netea M.G., Domínguez-Andrés J., Barreiro L.B., Chavakis T., Divangahi M., Fuchs E., Joosten L.A.B., van der Meer J.W.M., Mhlanga M.M., Mulder W.J.M. (2020). Defining trained immunity and its role in health and disease. Nat. Rev. Immunol..

[33] Saeed S., Quintin J., Kerstens H.H.D., Rao N.A., Aghajanirefah A., Matarese F., Cheng S.C., Ratter J., Berentsen K., van der Ent M.A. (2014). Epigenetic programming of monocyte-to-macrophage differentiation and trained innate immunity. Science.

[34] Hirschfeld M., Ma Y., Weis J.H., Vogel S.N., Weis J.J. (2000). Cutting edge: repurification of lipopolysaccharide eliminates signaling through both human and murine toll-like receptor 2. J. Immunol..

[35] Moorlag S.J.C.F.M., Folkman L., Ter Horst R., Krausgruber T., Barreca D., Schuster L.C., Fife V., Matzaraki V., Li W., Reichl S. (2024). Multi-omics analysis of innate and adaptive responses to BCG vaccination reveals epigenetic cell states that predict trained immunity. Immunity.

[36] Kromhout D., Spaaij C.J.K., de Goede J., Weggemans R.M. (2016). The 2015 Dutch food-based dietary guidelines. Eur. J. Clin. Nutr..

[37] United Nation University W.H.O., Food and Agriculture organization of the United Nations (2001).

[38] de Bree L.C.J., Mourits V.P., Koeken V.A., Moorlag S.J., Janssen R., Folkman L., Barreca D., Krausgruber T., Fife-Gernedl V., Novakovic B. (2020). Circadian rhythm influences induction of trained immunity by BCG vaccination. J. Clin. Invest..

[39] Alarcon-Barrera J.C., von Hegedus J.H., Brouwers H., Steenvoorden E., Ioan-Facsinay A., Mayboroda O.A., Ondo-Mendez A., Giera M. (2020). Lipid metabolism of leukocytes in the unstimulated and activated states. Anal. Bioanal. Chem..

[40] Weber C., Shantsila E., Hristov M., Caligiuri G., Guzik T., Heine G.H., Hoefer I.E., Monaco C., Peter K., Rainger E. (2016). Role and analysis of monocyte subsets in cardiovascular disease. Joint consensus document of the European Society of Cardiology (ESC) Working Groups "Atherosclerosis & Vascular Biology" and "Thrombosis". Thromb. Haemost..

[41] Ziegler-Heitbrock L., Ancuta P., Crowe S., Dalod M., Grau V., Hart D.N., Leenen P.J.M., Liu Y.J., MacPherson G., Randolph G.J. (2010). Nomenclature of monocytes and dendritic cells in blood. Blood.

[42] Bekkering S., Blok B.A., Joosten L.A.B., Riksen N.P., van Crevel R., Netea M.G. (2016). In Vitro Experimental Model of Trained Innate Immunity in Human Primary Monocytes. Clin. Vaccine Immunol..

[43] Dominguez-Andres J., Arts R.J.W., Bekkering S., Bahrar H., Blok B.A., de Bree L.C.J., Bruno M., Bulut O., Debisarun P.A., Dijkstra H. (2021). In vitro induction of trained immunity in adherent human monocytes. STAR Protoc.

[44] Franssen R., Schimmel A.W., van Leuven S.I., Wolfkamp S.C., Stroes E.S., Dallinga-Thie G.M. (2012). In Vivo Inflammation Does Not Impair ABCA1-Mediated Cholesterol Efflux Capacity of HDL. Cholesterol.

