# Supplementary material for: Single high-fat challenge and trained innate immunity: A randomized controlled cross-over trial
Source: iScience. 2024 Oct 5;27(11):111103. doi: 10.1016/j.isci.2024.111103 (PMC11530819; doi:10.1016/j.isci.2024.111103)
Supplement: Document S1. Figures S1–S6, Tables S1, and S2 [file mmc1.pdf]

iScience, Volume *27*

Supplemental information

Single high-fat challenge and trained innate immunity: A randomized controlled cross-over trial

Julia van Tuijl, Julia I.P. van Heck, Harsh Bahrar, Wieteke Broeders, Johan Wijma, Yvonne M. ten Have, Martin Giera, Heidi Zweers-van Essen, Laura Rodwell, Leo A.B. Joosten, Mihai G. Netea, Lydia A. Afman, Siroon Bekkering, and Niels P. Riksen

Supplemental information

Supplementary figures

Figure S1. Flow diagram of the randomization process. Related to Figure 1.

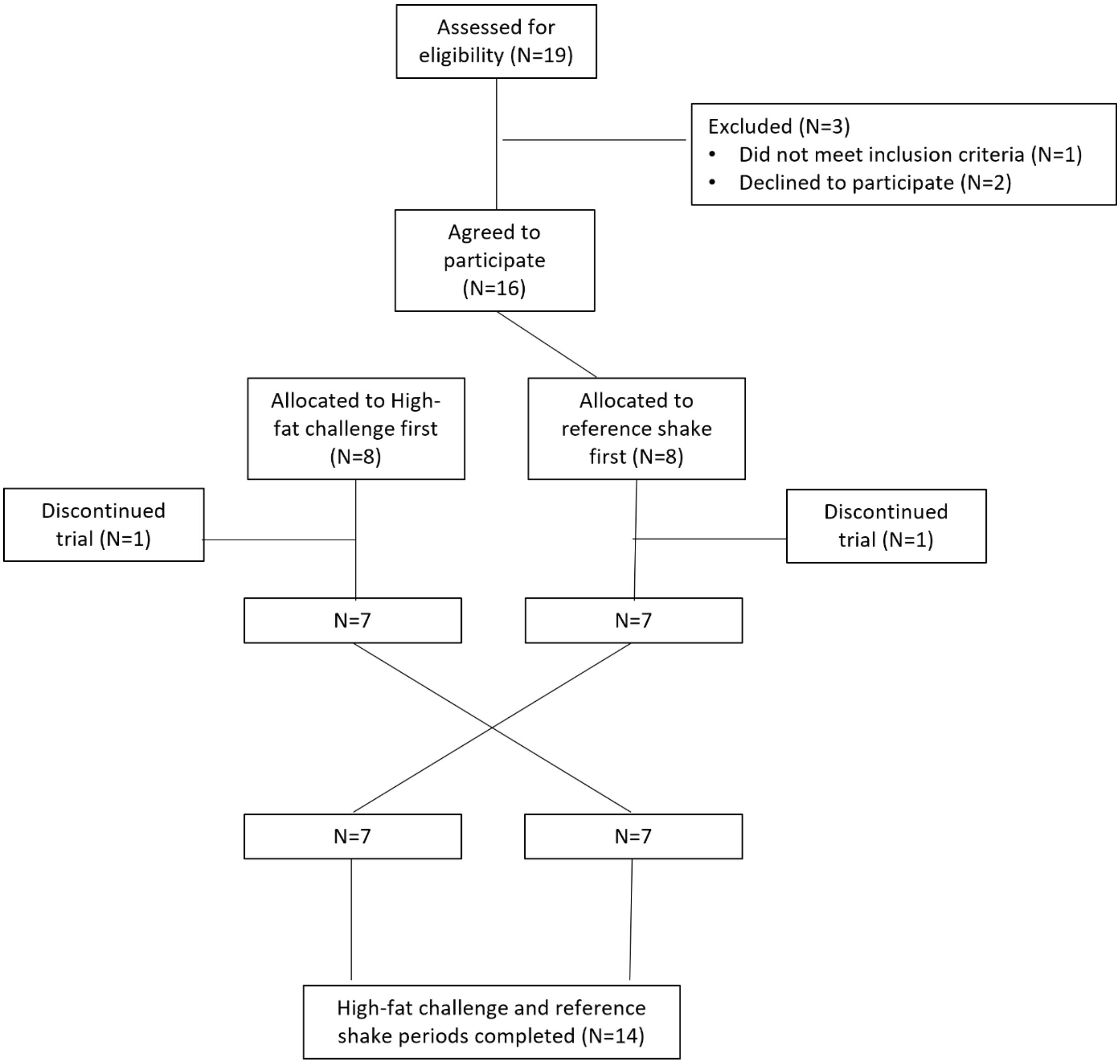

Figure S2

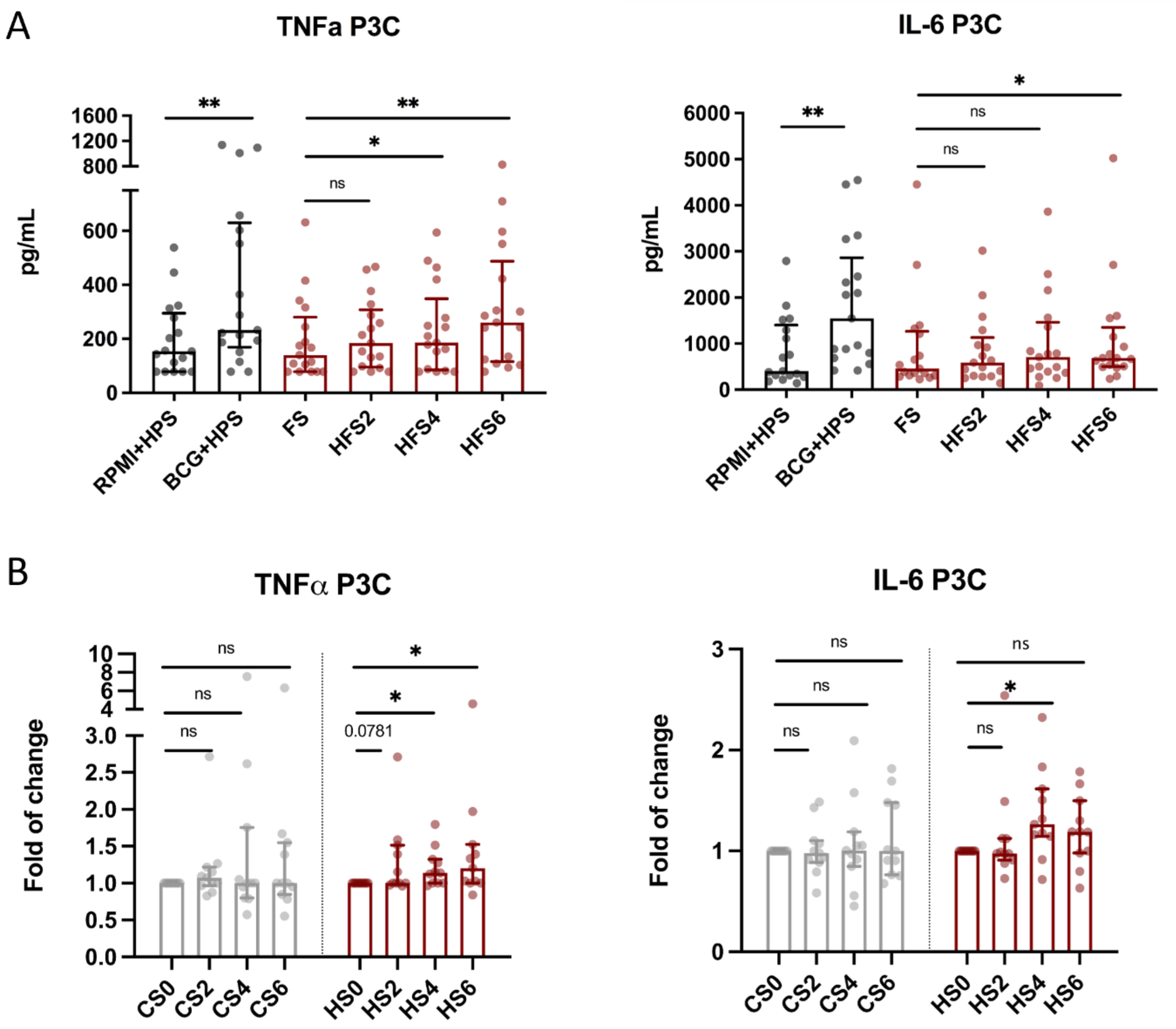

Figure S2, related to Figure 2. Exposure to serum obtained after a high-fat shake, but not after a common breakfast shake, induces trained immunity in healthy human monocytes. **A.** Serum obtained after the high-fat shake increased TNFα and IL-6 production of human monocyte-derived macrophages upon secondary stimulation with P3C, with the highest cytokine production after stimulation with serum obtained at t=6h (n=17). **B.** Cytokine production capacity was measured after 24h exposure to reference shake serum (CS) and the high-fat shake serum (HFS). Data are presented as fold of change to the fasting serum obtained before consumption of the control shake (CS0) or the high-fat shake (HFS0) (n=11). Median ±IQR. * indicates $p < 0.05$, ** $p < 0.01$, Wilcoxon-signed rank test.

Figure S3

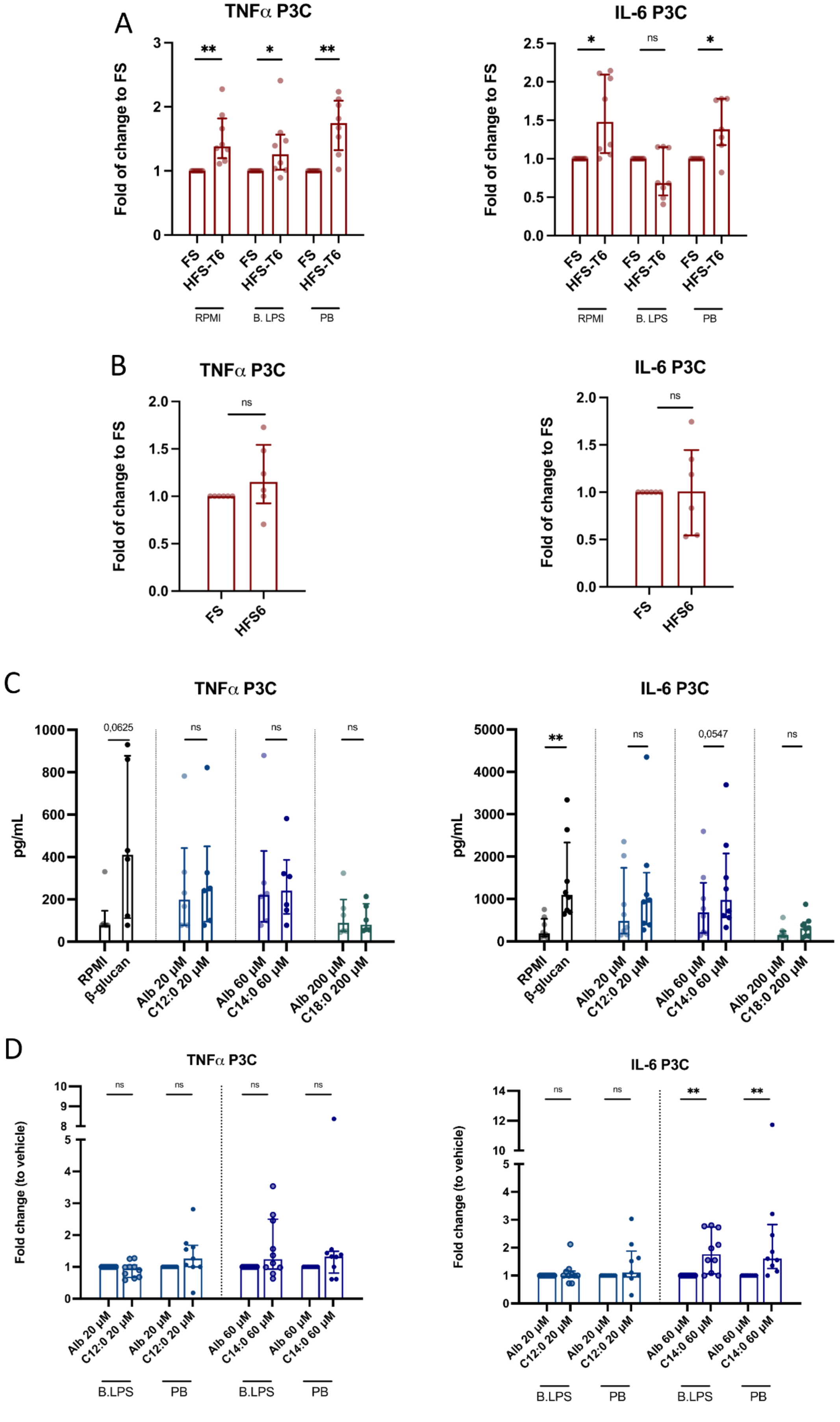

A
TNFα P3C
IL-6 P3C
Fold of change to FS
FS
HFS-T6
RPMI
B. LPS
PB
**
*
ns
B
TNFα P3C
IL-6 P3C
FS
HFS6
ns
C
TNFα P3C
IL-6 P3C
pg/mL
0,0625
0,0547
RPMI
β-glucan
Alb 20 µM
C12:0 20 µM
Alb 60 µM
C14:0 60 µM
Alb 200 µM
C18:0 200 µM
D
TNFα P3C
IL-6 P3C
Fold change (to vehicle)
B.LPS
PB

Figure S3, related to Figure 3. High-fat shake induced trained immunity is regulated via TLR4, but is not mediated via low-dose LPS, triglyceride-rich lipoproteins or the saturated fatty acids that are particularly increased after the high-fat shake. **A.** Adherent human monocytes were pre-incubated for 1 hour with plain RPMI or Bartonella LPS (B. LPS). After pre-incubation, the cells were exposed for 24 hours to high-fat serum obtained at t=0h (HFS0) or at t=6h (HFS6), with (PB) or without neutralization of LPS. On day 6, cells were restimulated with P3C for 24 hours and cytokine production was measured (n=8). Data are presented as fold of change to HFS0 for each separate inhibitor. **B.** HFS0 and HFS6 were depleted from apoB-containing lipoproteins before use in the training experiments (n=6). **C.** Monocytes were stimulated for 24 hours with albumin-conjugated C12:0, C14:0 and C18:0 or with the albumin vehicle (Alb) alone. After resting and differentiation, cytokine production upon restimulation was measured (TNFα n=6, IL-6 n=8). **D.** The same inhibition experiments as described under A. were performed for C12:0 and C14:0. Data are presented as fold of change to the vehicle control for each separate inhibitor (B. LPS n=10, PB n=9). Median ±IQR. * indicates $p < 0.05$, ** $p < 0.01$, Wilcoxon-signed rank test. CS = serum after the reference shake; HFS = serum after the high fat shake.

Figure S4

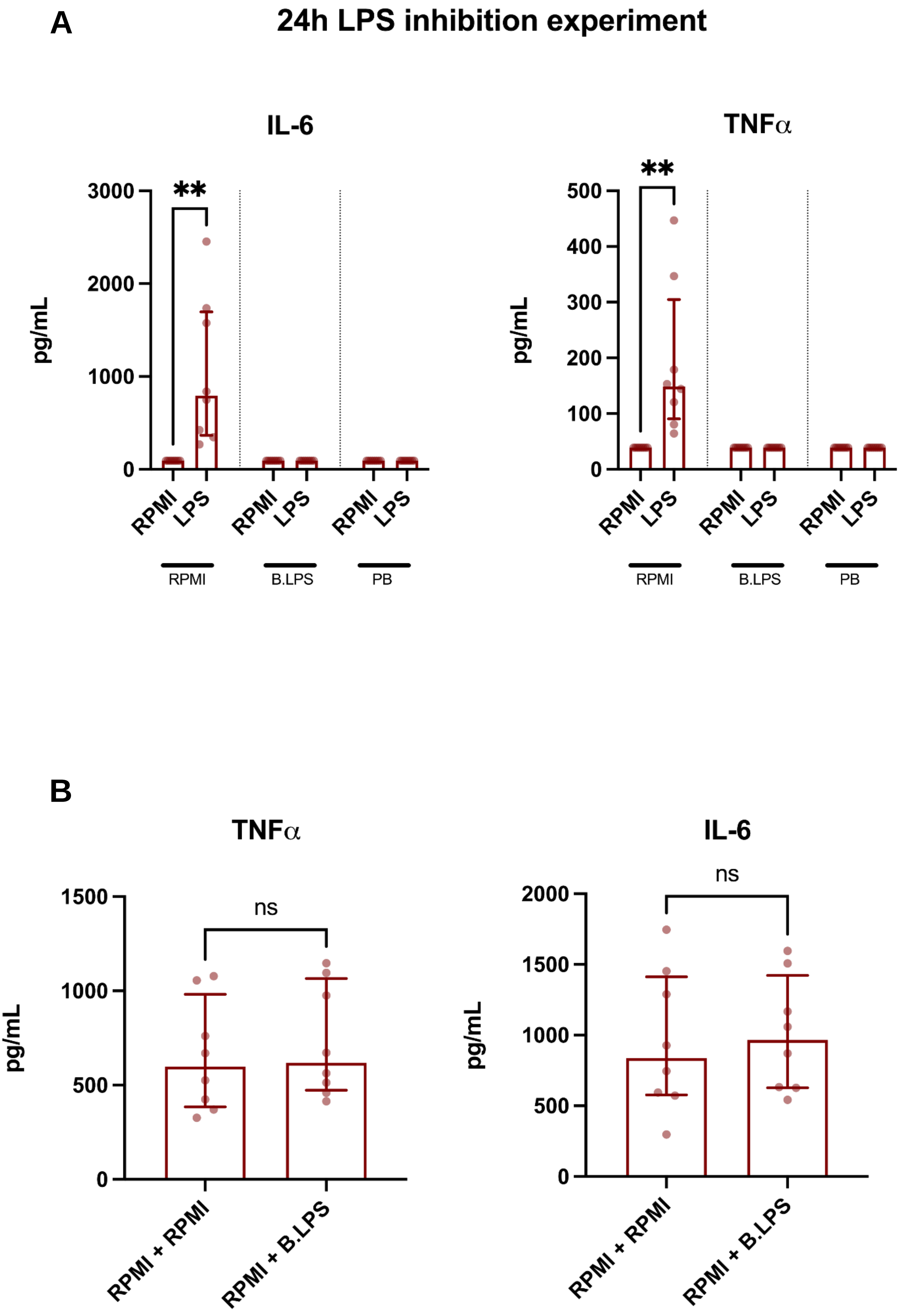
A
24h LPS inhibition experiment
IL-6
TNFα
pg/mL
3000
2000
1000
0
500
400
300
200
100
**
RPMI
LPS
RPMI
B.LPS
PB
B
TNFα
IL-6
1500
ns
RPMI + RPMI
RPMI + B.LPS

Figure S4, related to Figure 3. A. Adherent human monocytes were pre-incubated for 1 hour with plain RPMI or Bartonella LPS (B. LPS). After pre-incubation, the cells were exposed for 24 hours to E. Coli LPS, with (PB) or without neutralization of LPS. After 24 hours, cytokine production was measured (n=8). Data are presented as median ±IQR. **B.** Adherent human monocytes were pre-incubated for 1 hour with plain RPMI or Bartonella LPS (B. LPS). After pre-incubation, the cells were exposed for 24 hours to a plain RPMI. On day 6, cells were restimulated for 24 hours with LPS and cytokine production was measured (n=8). Median ±IQR. ** $p < 0.01$, Wilcoxon-signed rank test.

Figure S5

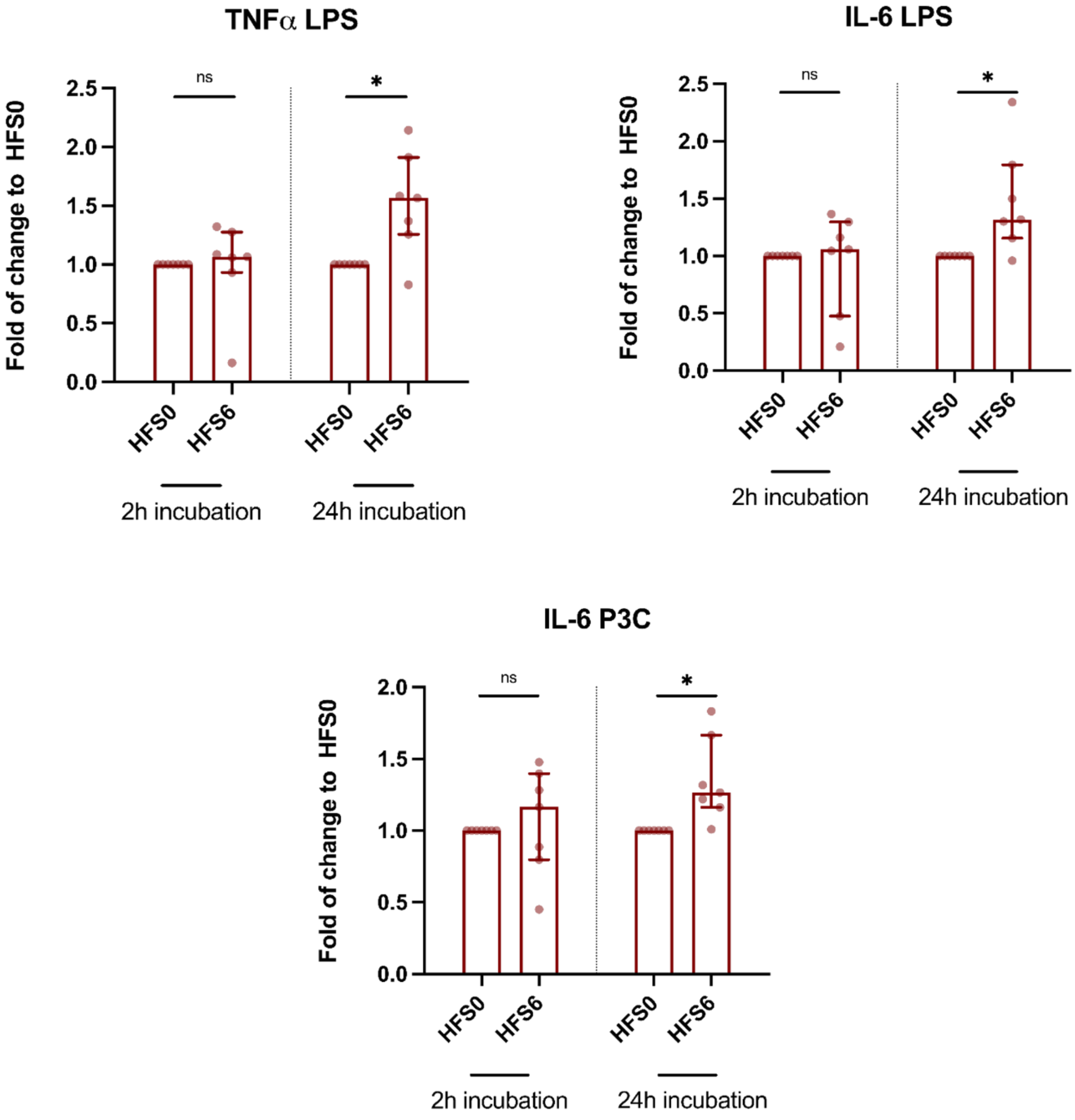

Figure S5. Adherent monocytes were incubated with the serum obtained at t=0h and t=6h after the high-fat shake for either 2 hours or 24 hours. On day 6, the cells were restimulated with LPS or P3C for 24 hours, after which cytokine production was measured (TNFα and IL-6). As most replicates were below detection limit for TNFα upon P3C restimulation, these results are excluded from the analysis. Data are presented as fold change of HFS6 over HFS0 (n=7). Median ±IQR. * indicates $p < 0.05$, Wilcoxon-signed rank test.

Figure S6

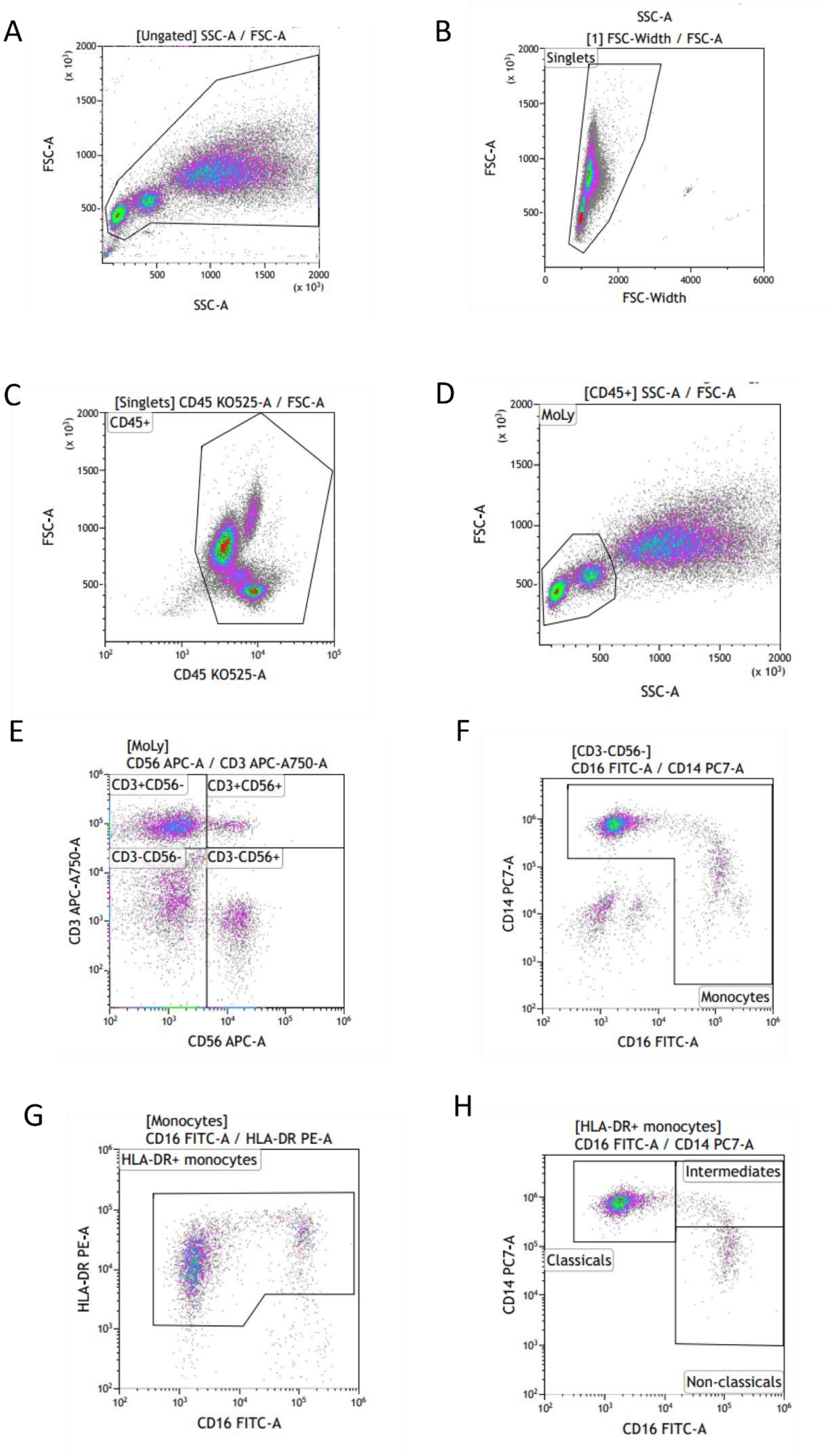

A
[Ungated] SSC-A / FSC-A
FSC-A
SSC-A
B
SSC-A
[1] FSC-Width / FSC-A
Singlets
FSC-A
FSC-Width
C
[Singlets] CD45 KO525-A / FSC-A
CD45+
FSC-A
CD45 KO525-A
D
[CD45+] SSC-A / FSC-A
MoLy
FSC-A
SSC-A
E
[MoLy]
CD56 APC-A / CD3 APC-A750-A
CD3+CD56-
CD3+CD56+
CD3-CD56-
CD3-CD56+
CD3 APC-A750-A
CD56 APC-A
F
[CD3-CD56-]
CD16 FITC-A / CD14 PC7-A
Monocytes
CD14 PC7-A
CD16 FITC-A
G
[Monocytes]
CD16 FITC-A / HLA-DR PE-A
HLA-DR+ monocytes
HLA-DR PE-A
CD16 FITC-A
H
[HLA-DR+ monocytes]
CD16 FITC-A / CD14 PC7-A
Intermediates
Classicals
Non-classicals
CD14 PC7-A
CD16 FITC-A

Figure S6, related to Table 2. Gating strategy for flow cytometry analysis. **A.** Debris was removed. **B.** Singlet were selected. **C.** Gating for CD45+ cells. **D.** Gating for monocytes and lymphocytes (MoLy) based on forward- and side scatter. **E.** The CD3-CD56 plot was used for the exclusion of T-lymphocytes and natural-killer cells. The CD3-CD56- quadrant was selected for further gating. **F.** B-cells were excluded based on low CD14 and low CD16 expression. **G.** HLA-DR- cells were excluded. **H.** In a CD14-CD16 plot the total monocyte population was divided into subsets: classical monocytes (CD14++CD16-), intermediate monocytes (CD14++CD16+) and non-classical monocytes (CD14+CD16++).

Supplementary tables

Table S1. Example of a standardized meal plan (2000 kcal)

Breakfast
- 2 slices of whole wheat bread with 1 unit (=10 grams) low-fat margarine, i.e. 1 with slim cheese and 1 with jam<br>- 250 mL of semi-skimmed milk
**Snack**
- 1 portion of fruit
**Lunch**
- 2 slices of whole wheat bread with 1 unit (=10 grams) low-fat margarine, i.e. 1 with ½ avocado and 1 with jam<br>- 150 mL of semi-skimmed milk
**Snack**
- 1 portion of fruit<br>- 25 grams of unsalted nuts
**Dinner**
- 4 serving spoons: potatoes<br>- 100 grams chicken<br>- 5 serving spoons: vegetables (250 g)<br>- 1 table spoon of olive oil for the preparation of the food

N.B. Please, do not drink any sodas, fruit juices or alcoholic beverages the day before or during study days. During study days it is allowed to drink tea and/or coffee (without the addition of sugar or milk) or water.

Total

Energy 1975 kcal:

- Fat 68.8 g (31 E%)

Of which saturated 17.6 g (8E%)

- Protein 94.3 g

- Carbohydrates 228.2 g (46 E%)

Of which sugars 88.3 g

- Fiber 31.6 g

- Salt 2.9 g

Substitute options

**Product**	**Substitute**
1 slice of bread	- 2 slices of knäckebröd<br>- 2 pieces of rusk<br>- 1 slice of rye bread<br>- 1 portion of oats (40 grams)<br>- 1 granola ball<br>- 1 slice of raisin bread
Savory sandwich toppings<br><br><br><br><br>Sweet sandwich toppings	- slim cheese<br>- hüttenkäse<br>- lean meat: chicken breast, turkey breast, roast beef<br><br>- jam<br>- apple syrup
1 portion of dairy	- 150 mL milk: (semi-)skimmed or buttermilk<br>- 150 mL soy milk<br>- 200 grams yoghurt: (semi-)skimmed<br>- 200 grams quark: (semi-)skimmed
1 portion of fruit	- 1 banana<br>- 1 apple<br>- 1 pear<br>- 1 orange<br>- 1 portion of grapes (125 grams)<br>- 2 peaches/apricots/prunes<br>- 2 clementines<br>- 2 kiwis
Drinks	- water<br>- tea, coffee (without sugar)<br>- sugar free lemonade
Meat/meat substitutes	- 100-125 grams chicken breast, turkey breast<br>- 100-125 grams lean beef<br>- 1-2 eggs<br>- 1 piece of vegetarian burger/balls, tofu, tempeh, etc.

Table S2

Nutritional value	High-fat shake	Reference shake
**Energy (kcal)**	900	400
**Protein (g)**	9	17
**Carbohydrates (g)**	22	49.5
**Fat (g)**	95	14.5
of which saturated (g)	54	9
**Fiber (g)**	0	2.3

Table S2. Overview of the nutrient composition of the high-fat and reference shake. Adapted from Esser et al.[1]

References

1. Esser D, Oosterink E, op 't Roodt J, Henry RM, Stehouwer CD, Muller M, Afman LA. Vascular and inflammatory high fat meal responses in young healthy men; a discriminative role of IL-8 observed in a randomized trial. *PLoS One* 2013;**8**:e53474.
